# Supplementary figures and images for: Δ133p53α and Δ160p53α isoforms of the tumor suppressor protein p53 exert dominant-negative effect primarily by co-aggregation
Source: eLife. 2025 Jul 21;14:RP106469. doi: 10.7554/eLife.106469 (PMC12279375; doi:10.7554/eLife.106469)

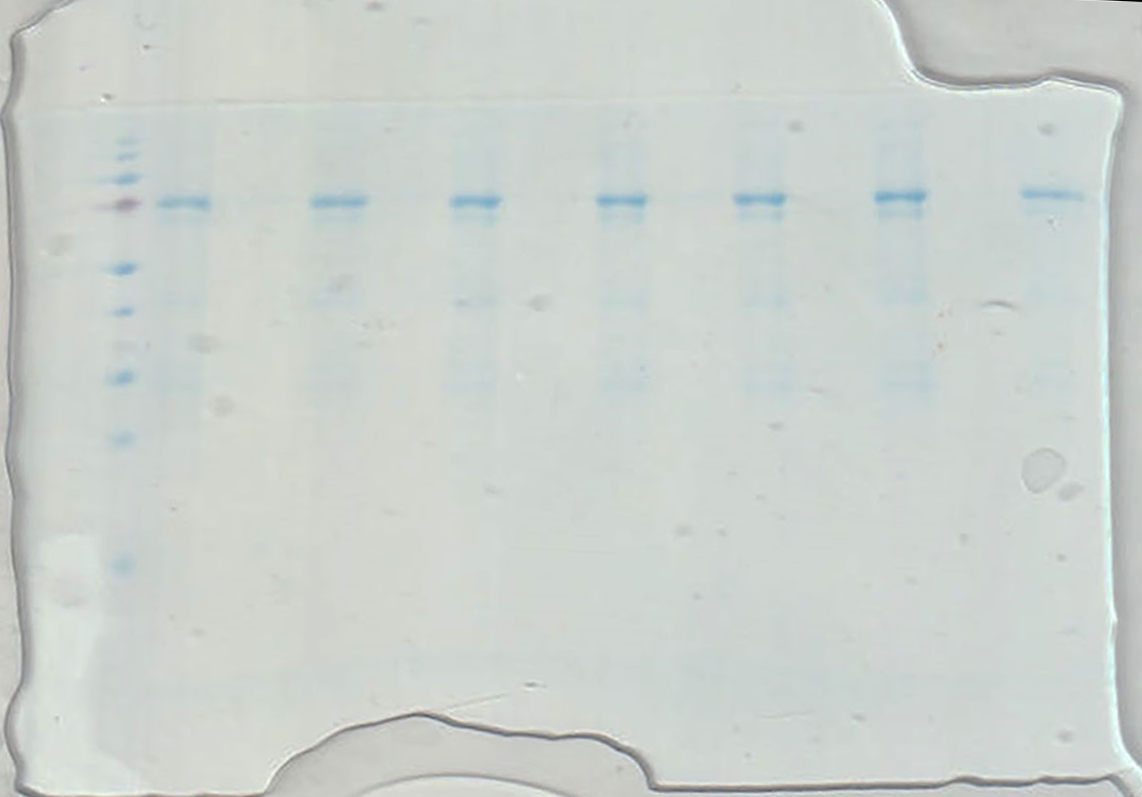

Supplement: Figure 1—figure supplement 2—source data 2. [file elife-106469-fig1-figsupp2-data2.zip › Figure 1—figure supplement 2_raw blot/FLp53.png]

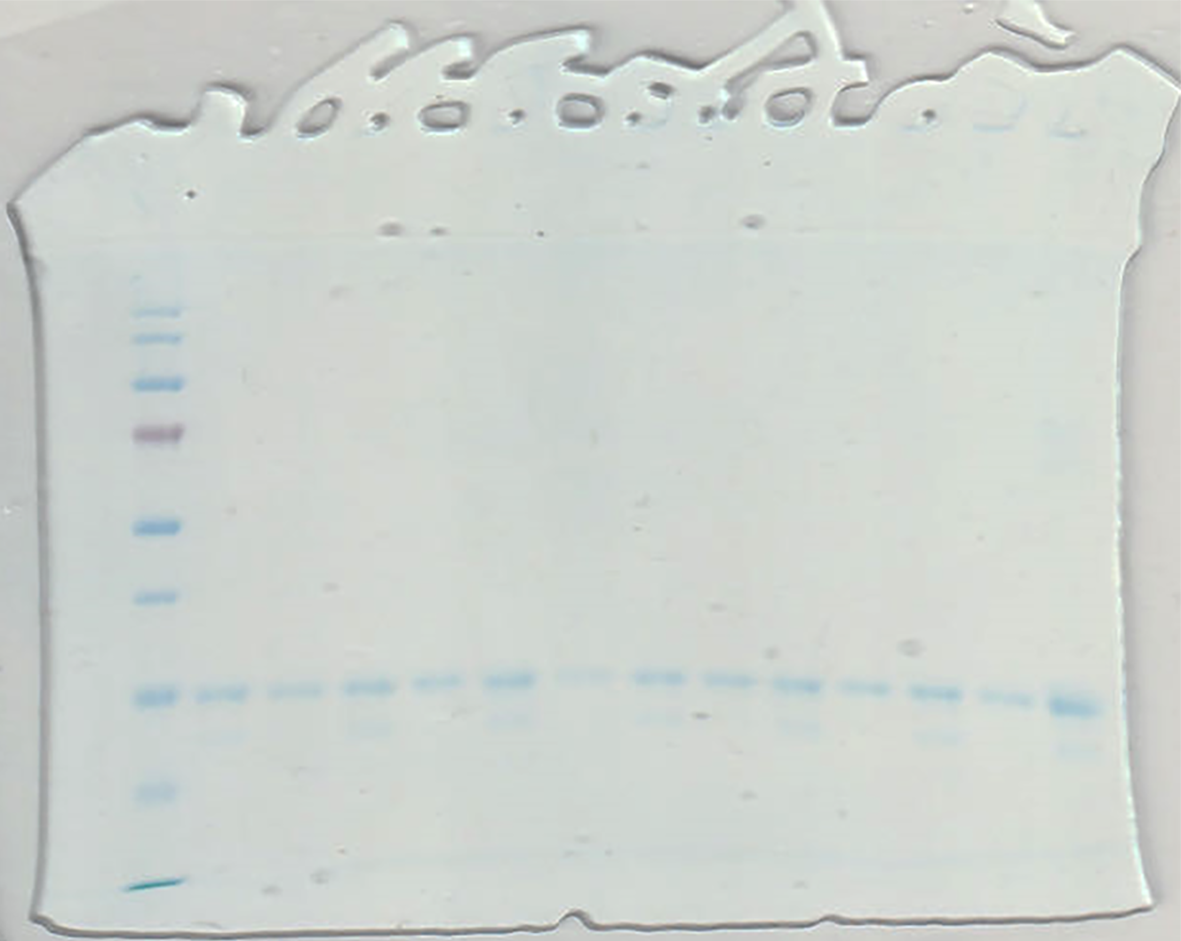

Supplement: Figure 1—figure supplement 2—source data 2. [file elife-106469-fig1-figsupp2-data2.zip › Figure 1—figure supplement 2_raw blot/delta133p53.png]

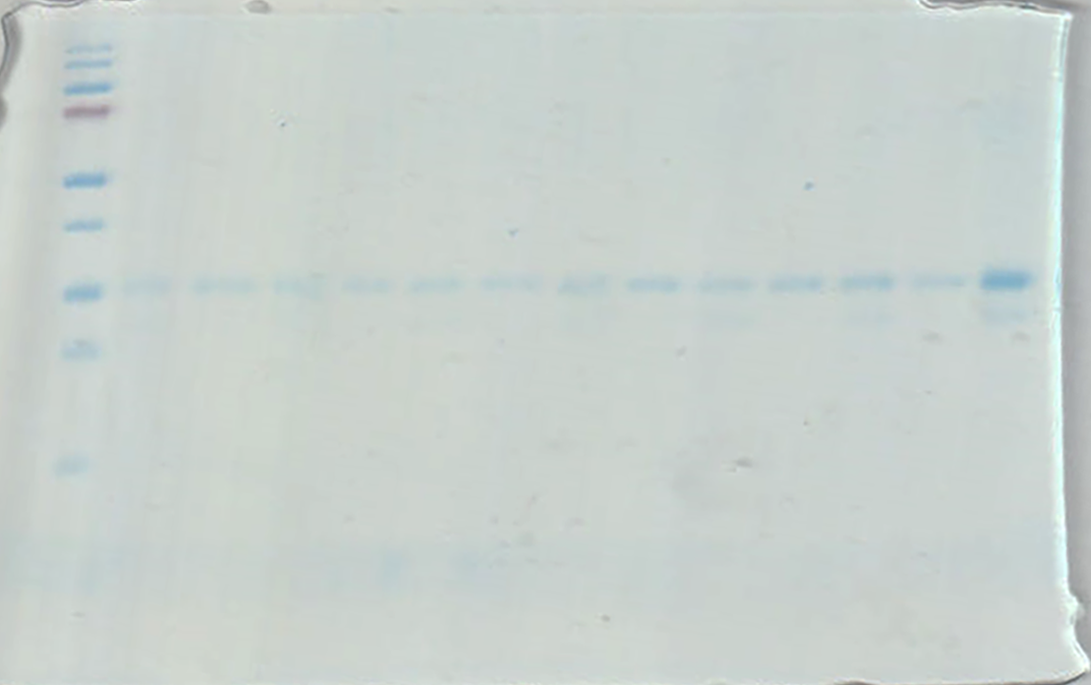

Supplement: Figure 1—figure supplement 2—source data 2. [file elife-106469-fig1-figsupp2-data2.zip › Figure 1—figure supplement 2_raw blot/delta160p53.png]

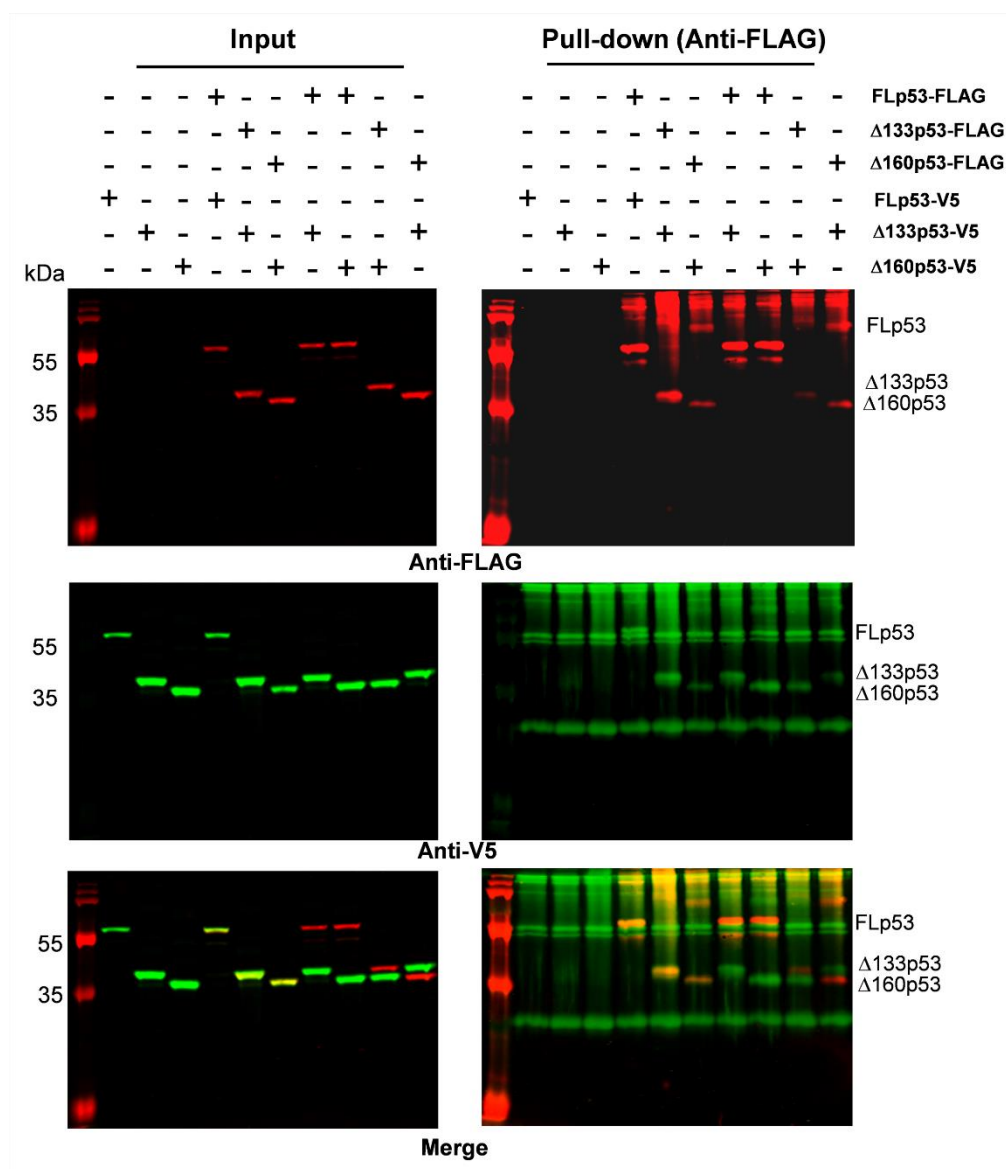

Figure 2-source data 1. Original membranes corresponding to Figure 2, panel A.

Supplement: Figure 2—source data 1. [file elife-106469-fig2-data1.zip › Figure 2-source data 1_labeled blot.pdf]

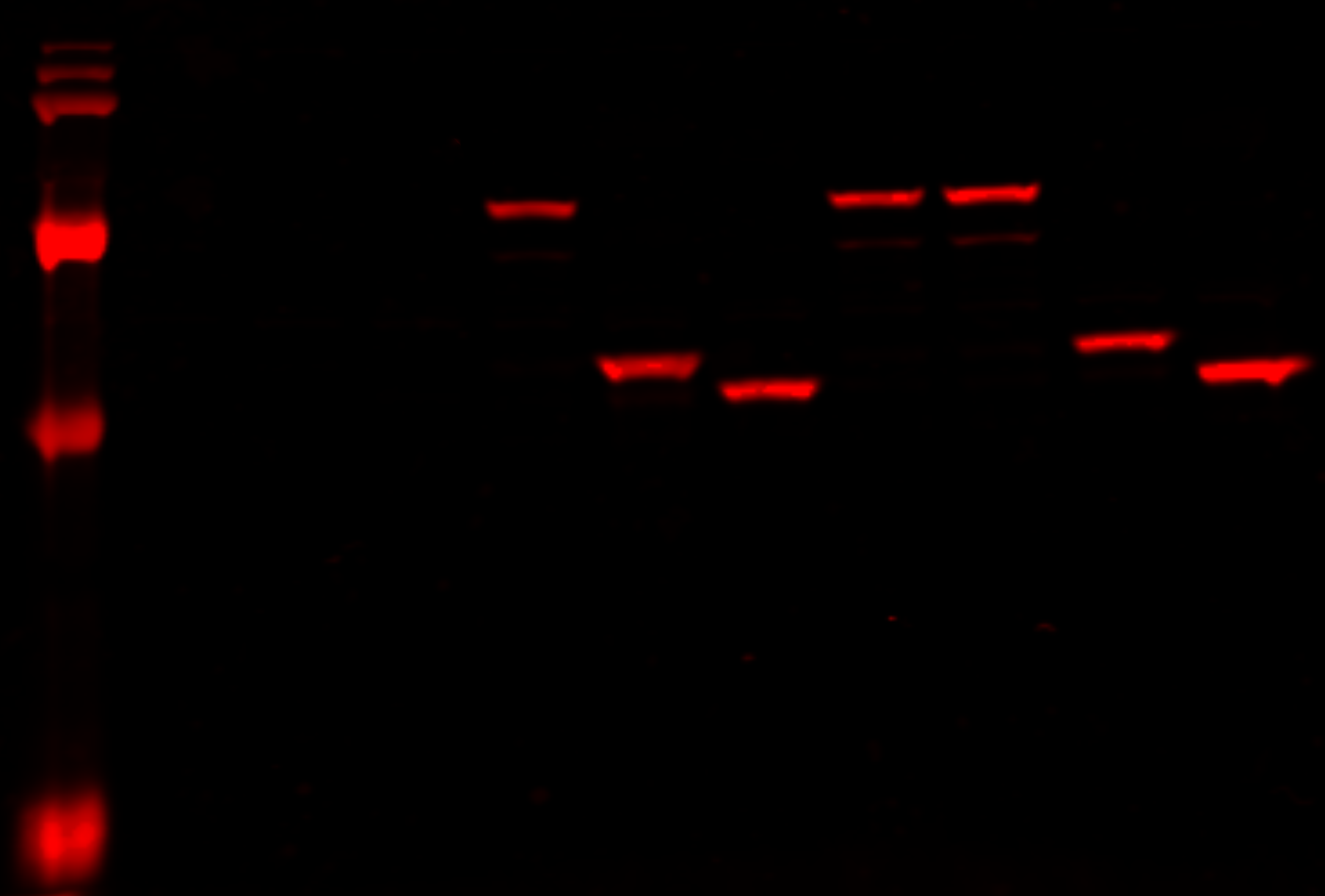

Supplement: Figure 2—source data 2. [file elife-106469-fig2-data2.zip › Figure 2_raw blot/Input_anti-FLAG.tif]

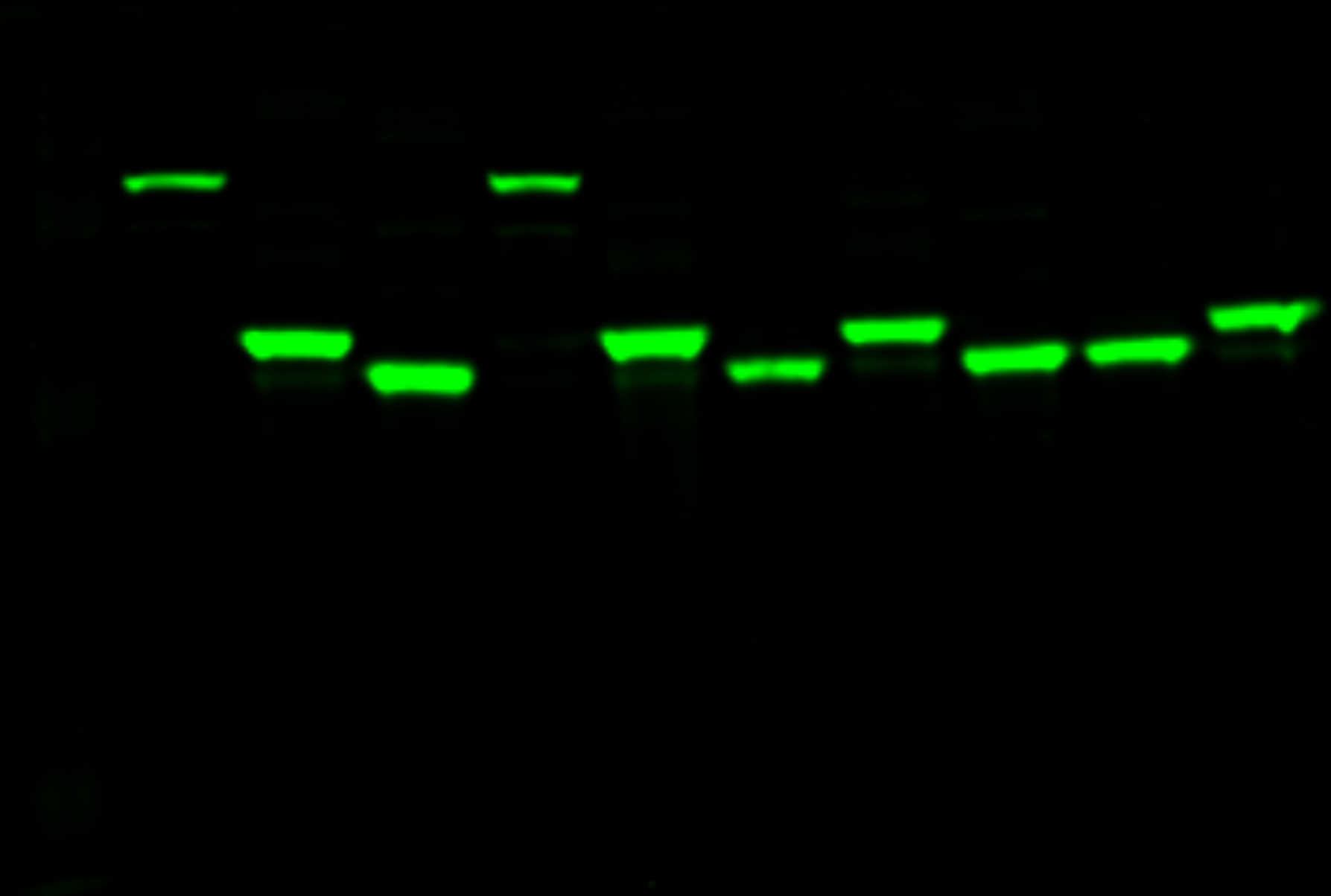

Supplement: Figure 2—source data 2. [file elife-106469-fig2-data2.zip › Figure 2_raw blot/Input_anti-V5.tif]

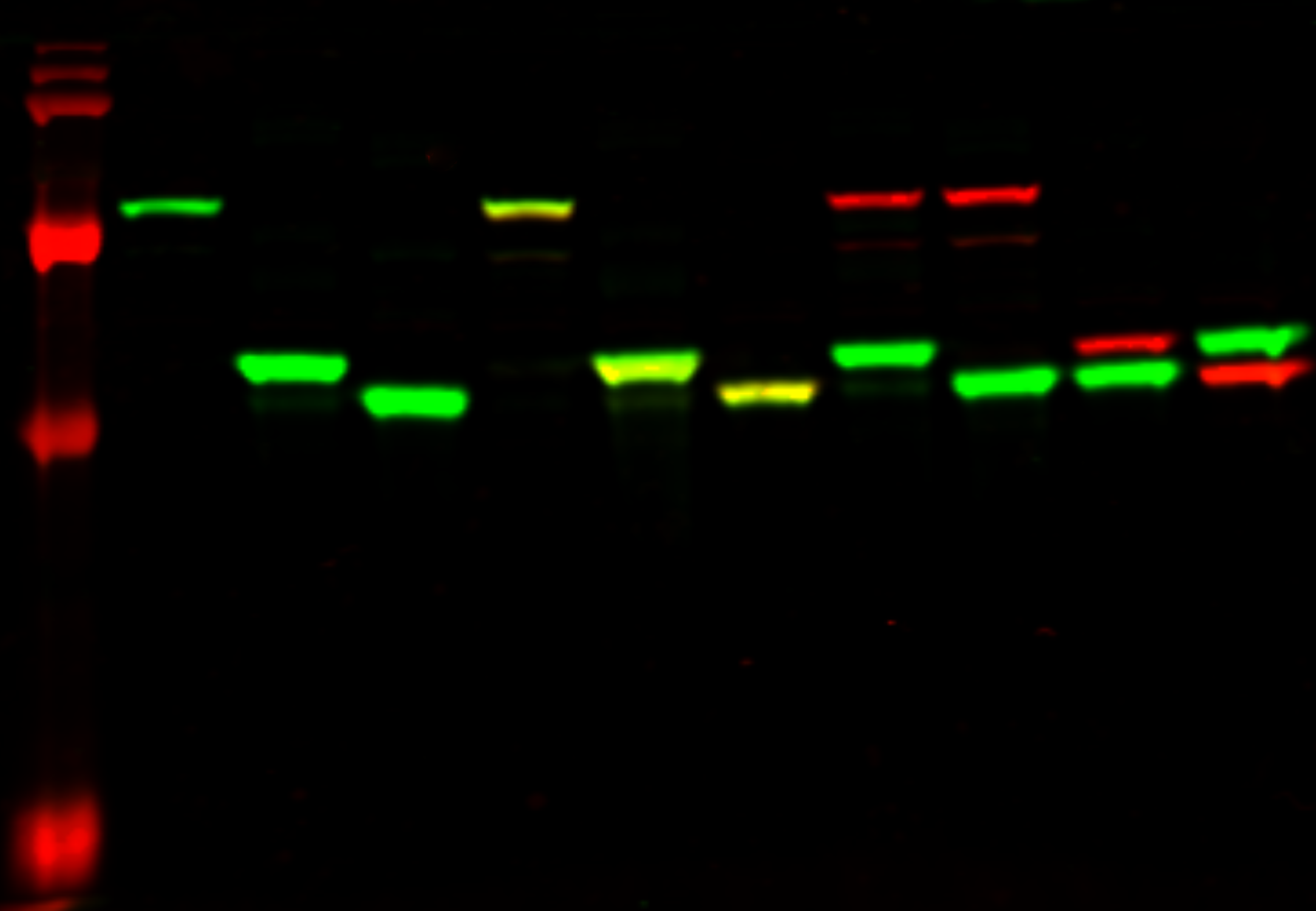

Supplement: Figure 2—source data 2. [file elife-106469-fig2-data2.zip › Figure 2_raw blot/Input_merge.tif]

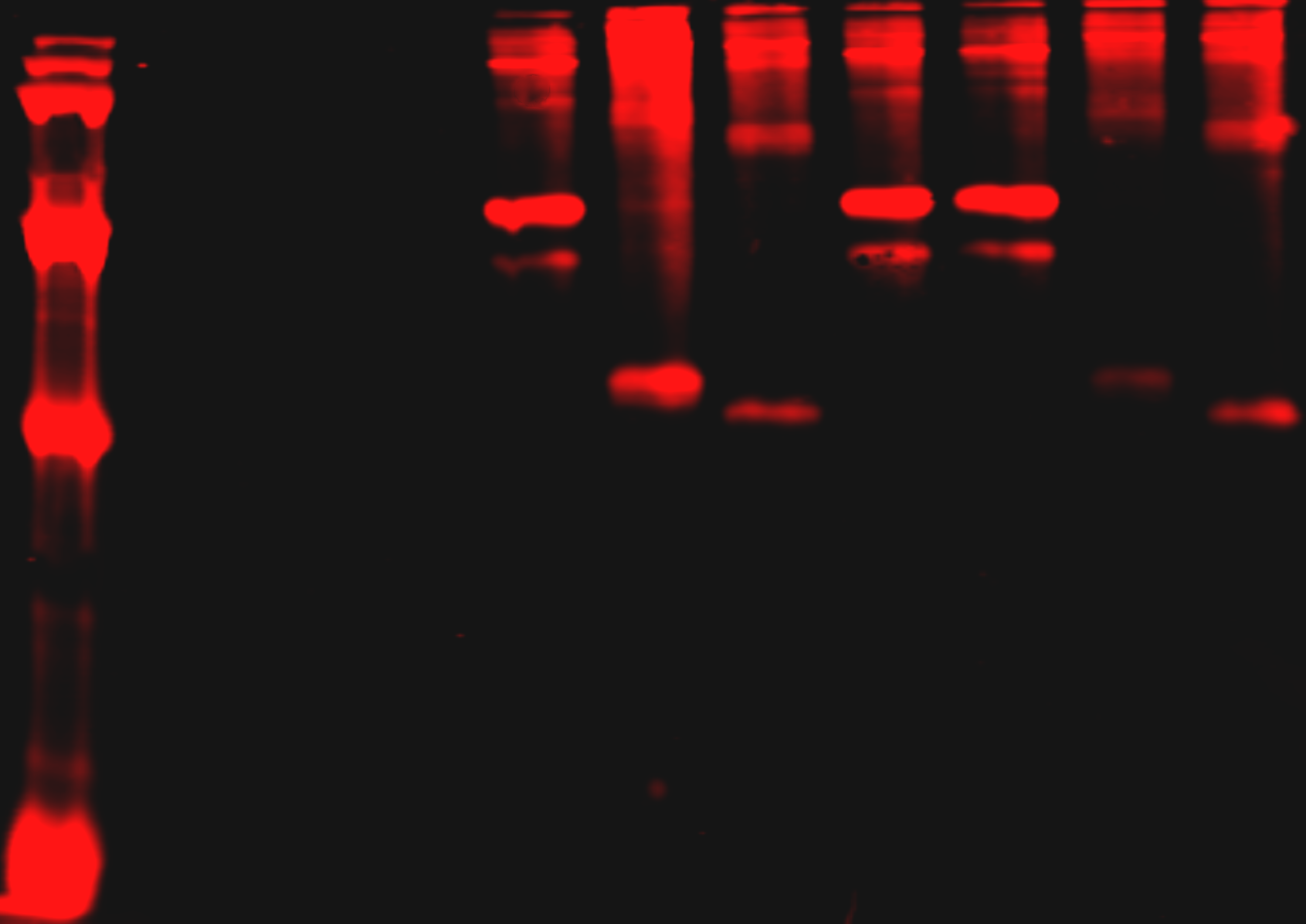

Supplement: Figure 2—source data 2. [file elife-106469-fig2-data2.zip › Figure 2_raw blot/Pull-down_anti-FLAG.tif]

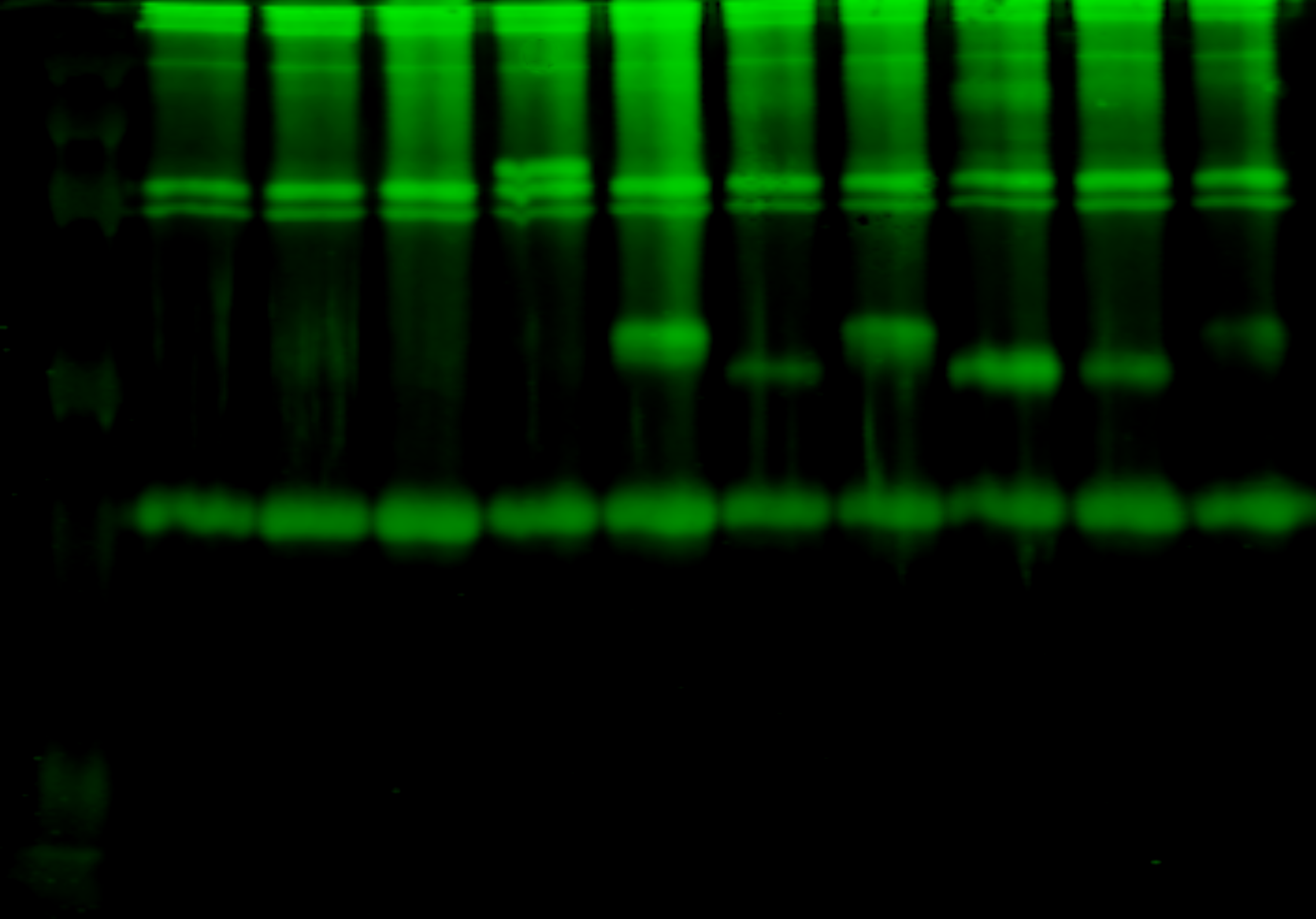

Supplement: Figure 2—source data 2. [file elife-106469-fig2-data2.zip › Figure 2_raw blot/Pull-down_anti-V5.tif]

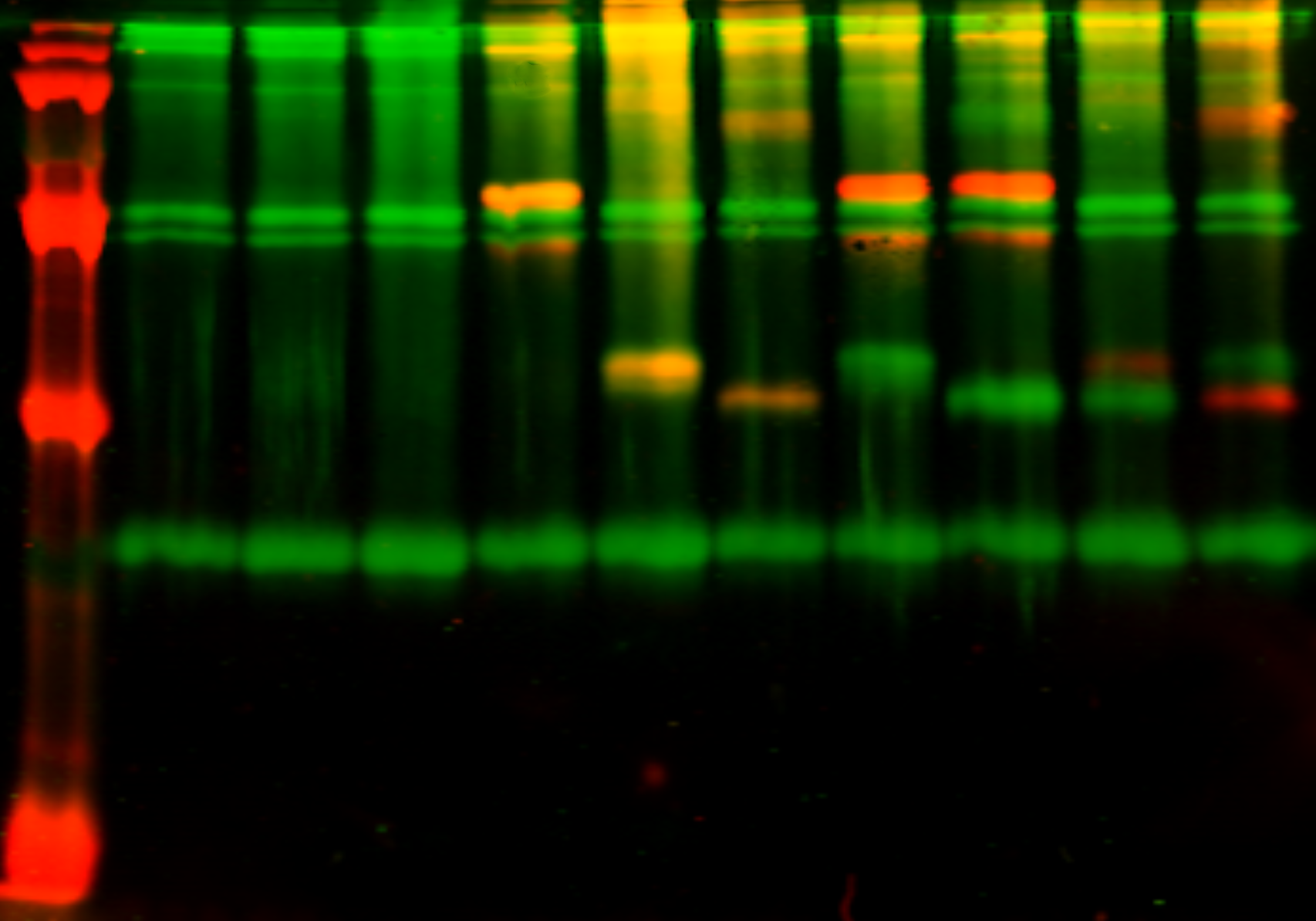

Supplement: Figure 2—source data 2. [file elife-106469-fig2-data2.zip › Figure 2_raw blot/Pull-down_merge.tif]

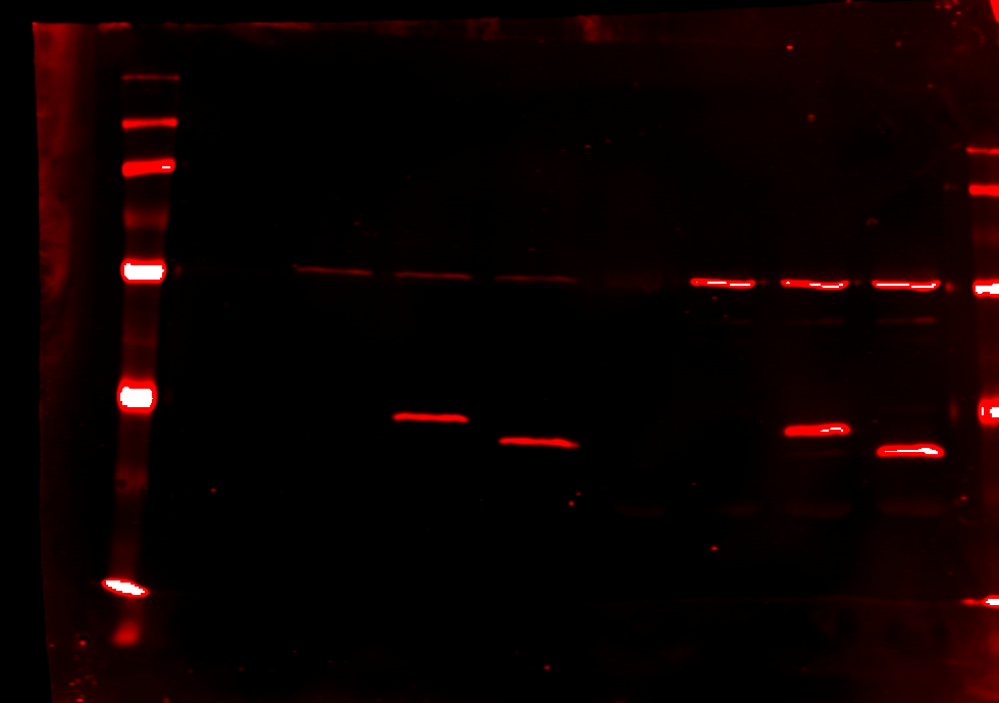

Supplement: Figure 2—figure supplement 1—source data 2. [file elife-106469-fig2-figsupp1-data2.zip › Figure 2—figure supplement 1_raw blot/Figure 2—figure supplement 1—source data 1_raw blot.png]

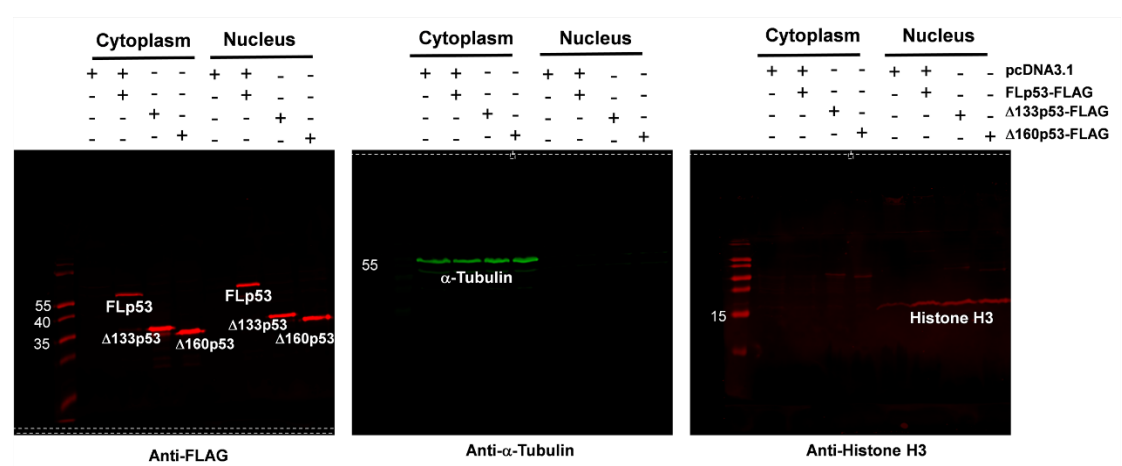

Figure 5-source data1. Original membranes corresponding to Figure 5, panel A.

Supplement: Figure 5—source data 1. [file elife-106469-fig5-data1.zip › Figure 5-source data 1_labeled blot.pdf]

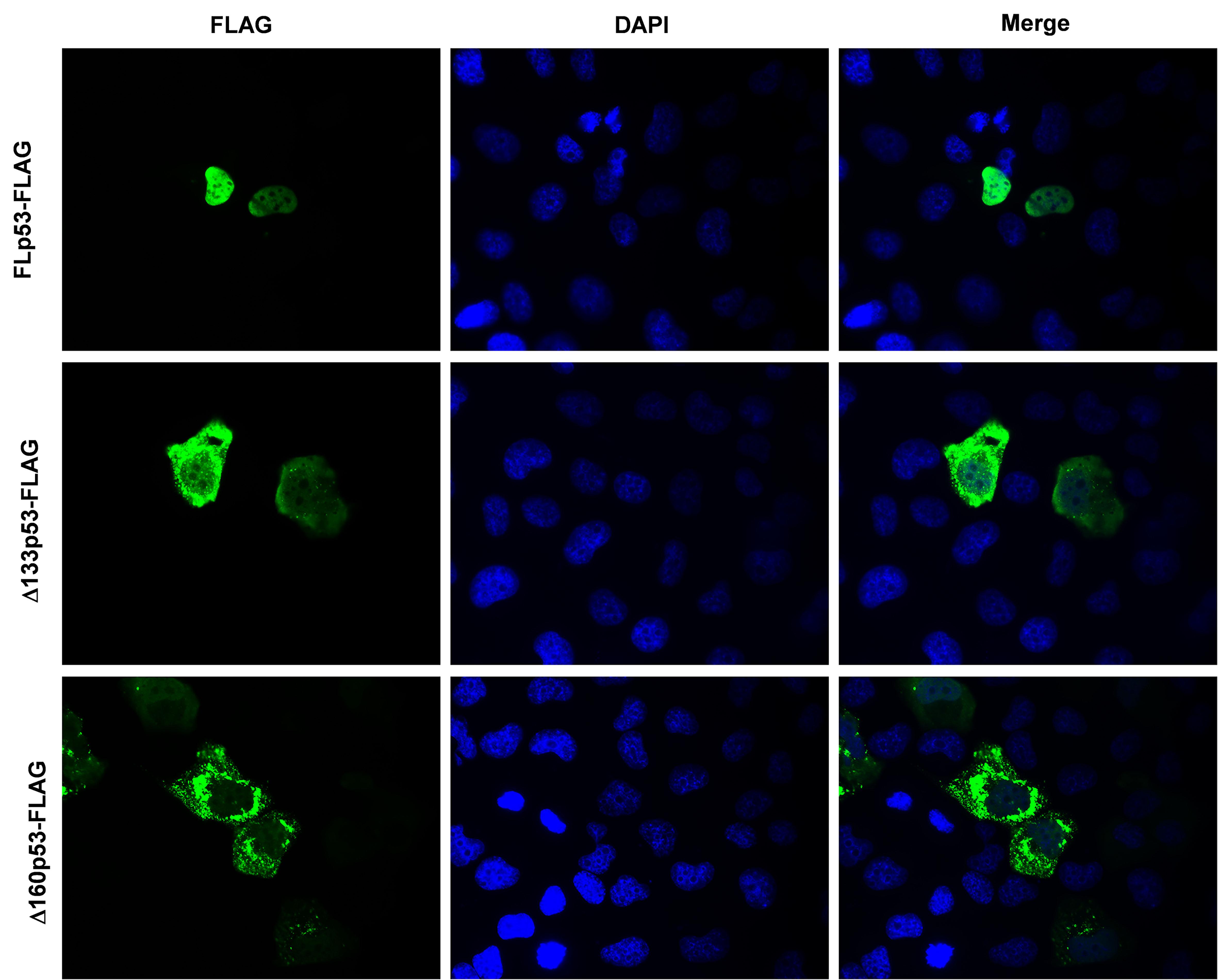

Supplement: Figure 5—source data 3. [file elife-106469-fig5-data3.jpg]

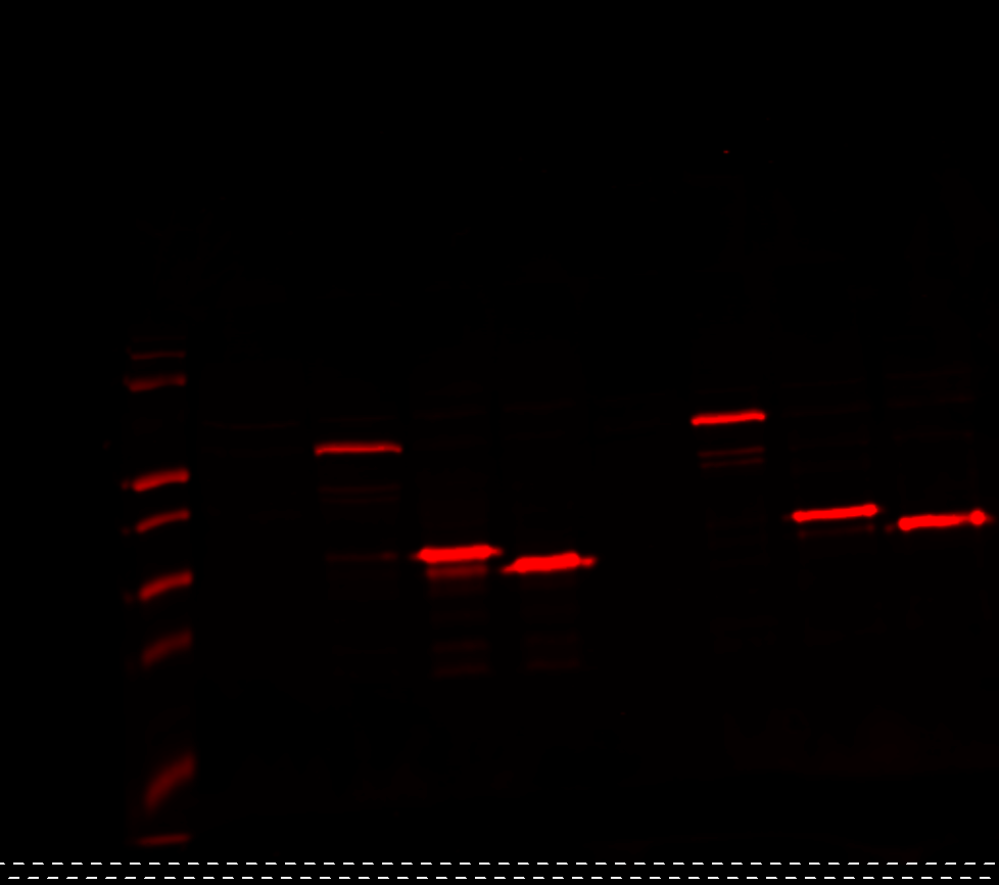

Supplement: Figure 5—source data 4. [file elife-106469-fig5-data4.zip › Figure 5_raw blot/Anti-FLAG.png]

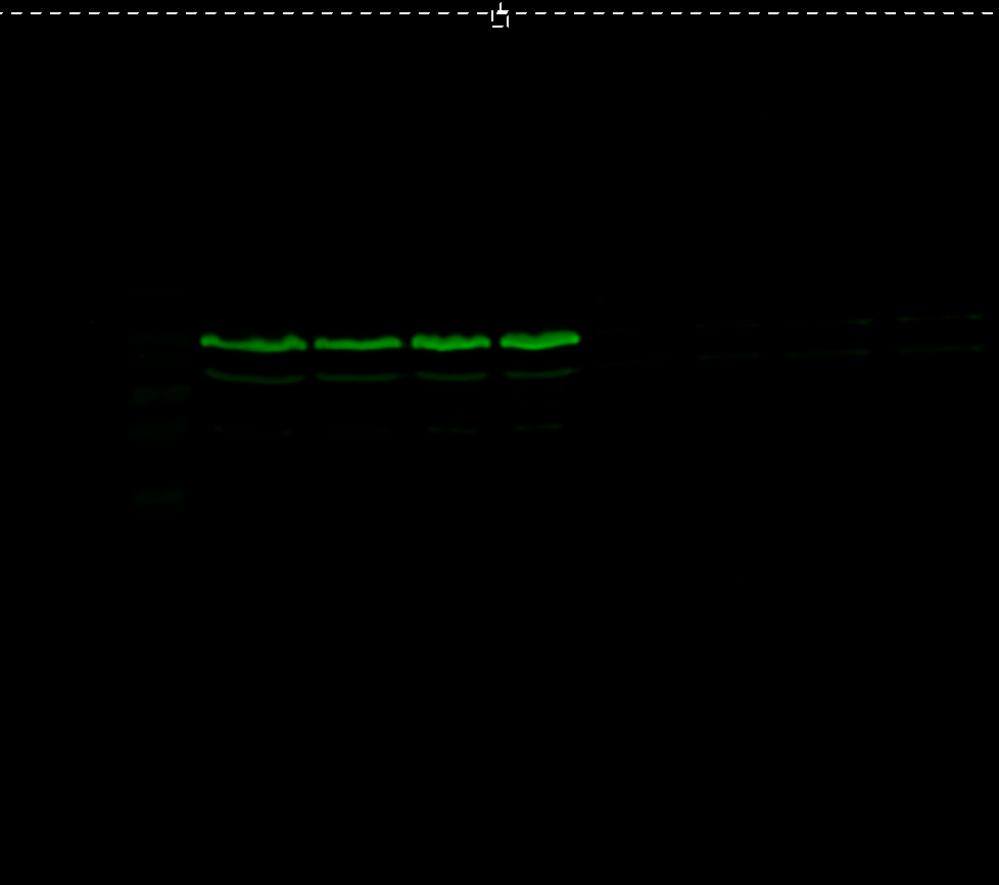

Supplement: Figure 5—source data 4. [file elife-106469-fig5-data4.zip › Figure 5_raw blot/Anti-Histone H3.png]

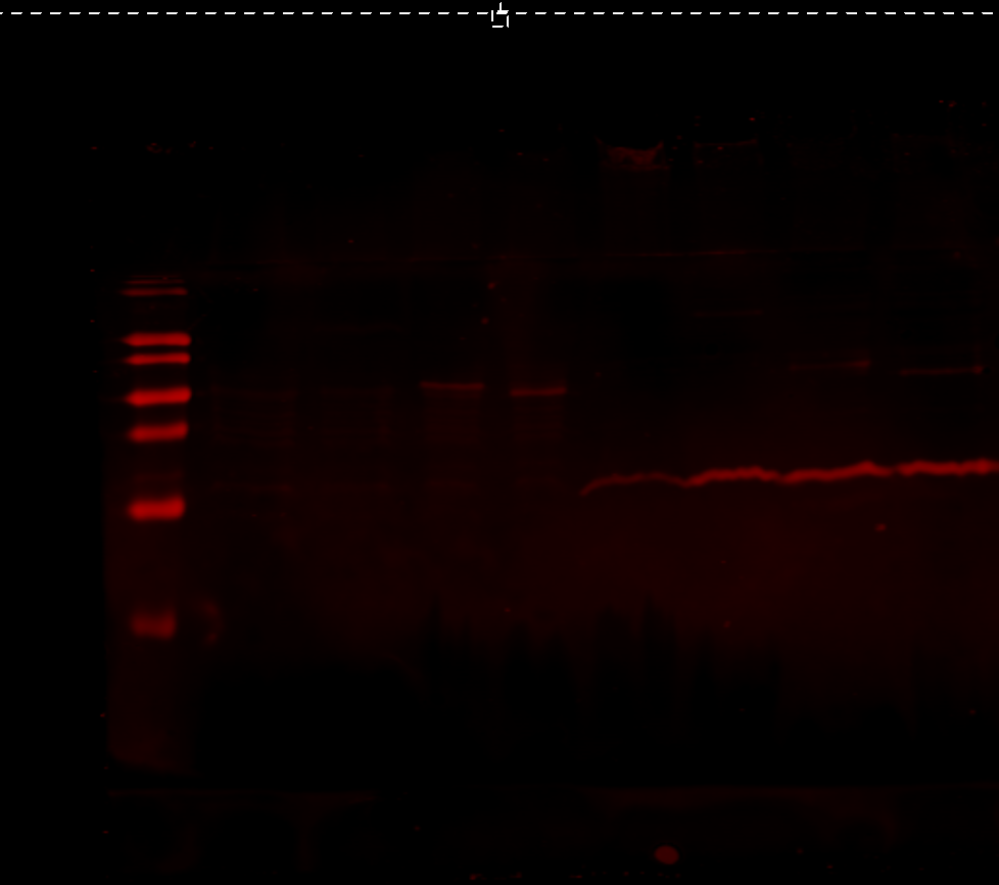

Supplement: Figure 5—source data 4. [file elife-106469-fig5-data4.zip › Figure 5_raw blot/Anti-Tubulin.png]

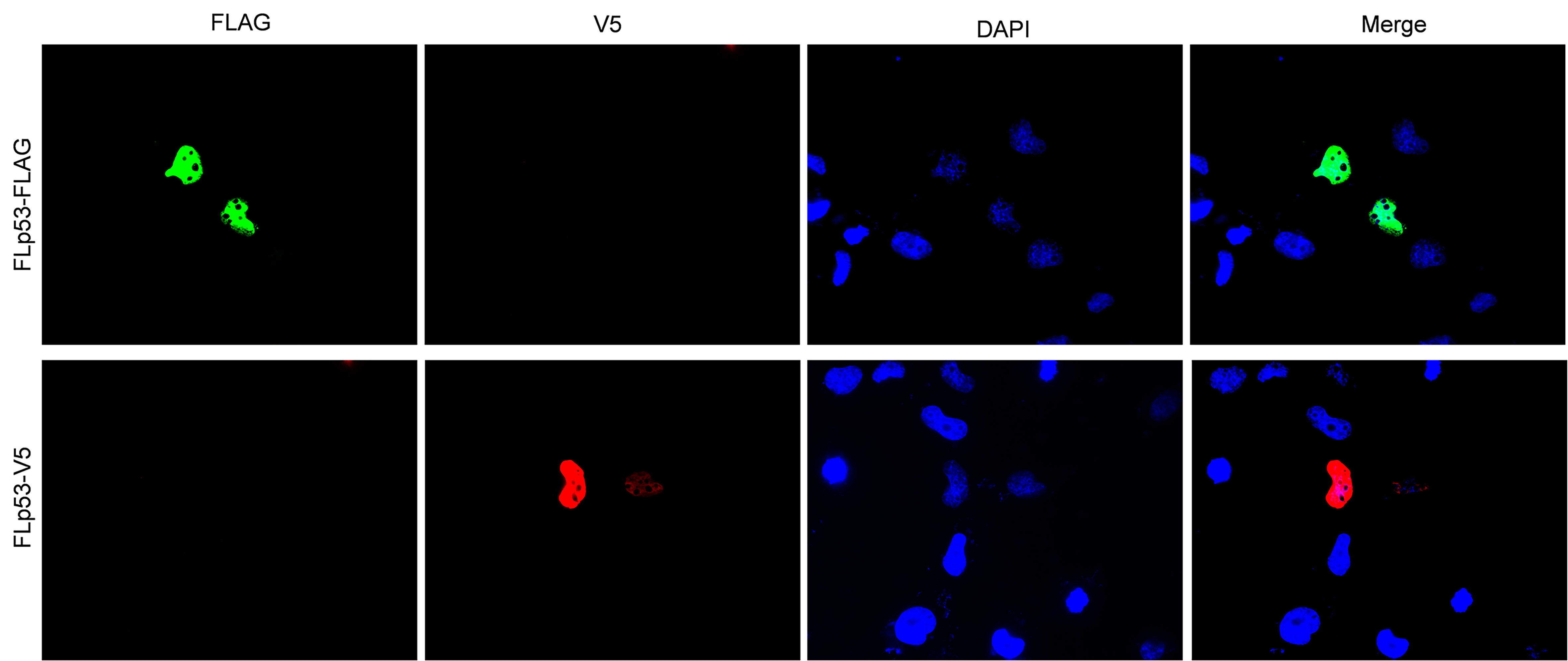

Supplement: Figure 5—figure supplement 1—source data 1. [file elife-106469-fig5-figsupp1-data1.jpg]

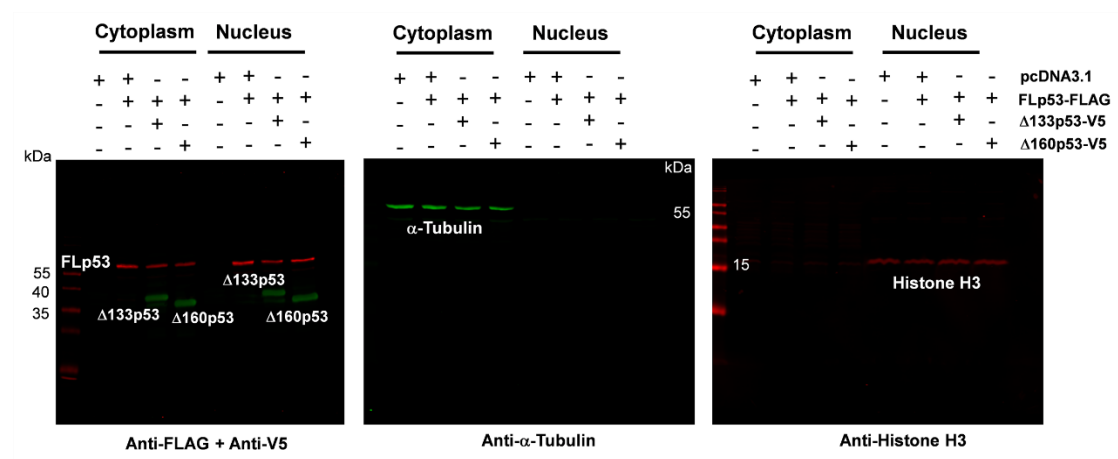

Figure 6-source data 1. Original membranes corresponding to Figure 6, panel A.

Supplement: Figure 6—source data 1. [file elife-106469-fig6-data1.zip › Figure 6-souce data 1_labeled blot.pdf]

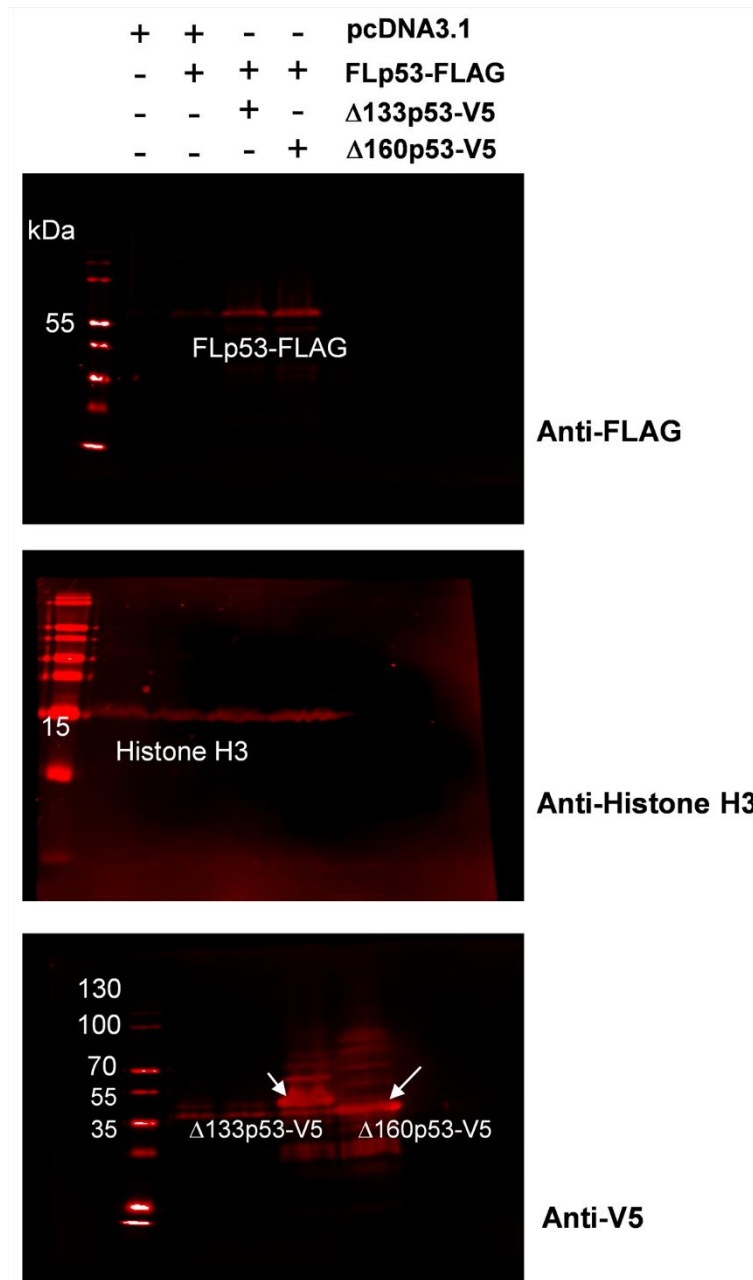

Figure 6-source data 2. Original membranes corresponding to Figure 6, panel B.

Supplement: Figure 6—source data 2. [file elife-106469-fig6-data2.zip › Figure 6-source data 2_labeled blot.pdf]

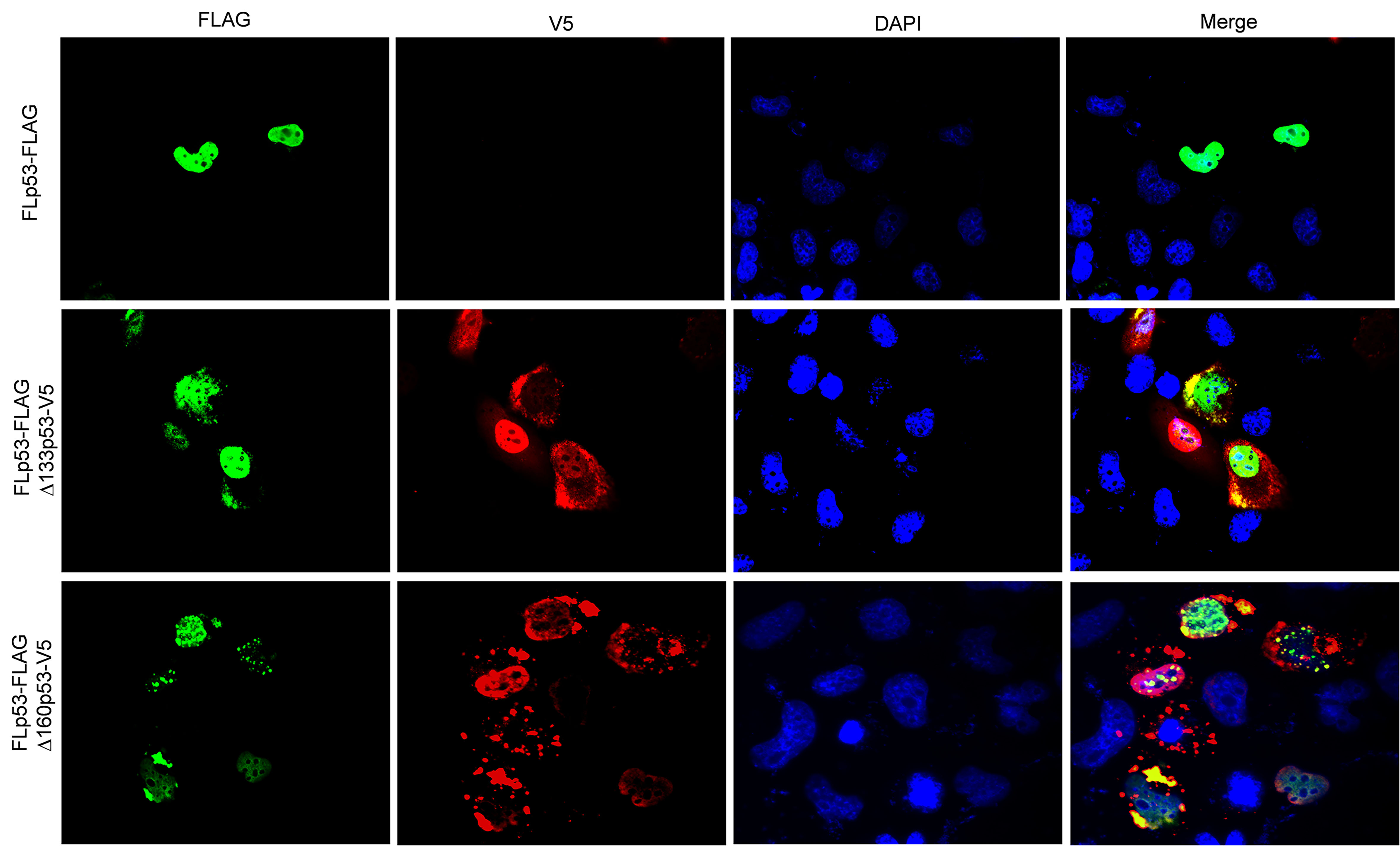

Supplement: Figure 6—source data 5. [file elife-106469-fig6-data5.jpg]

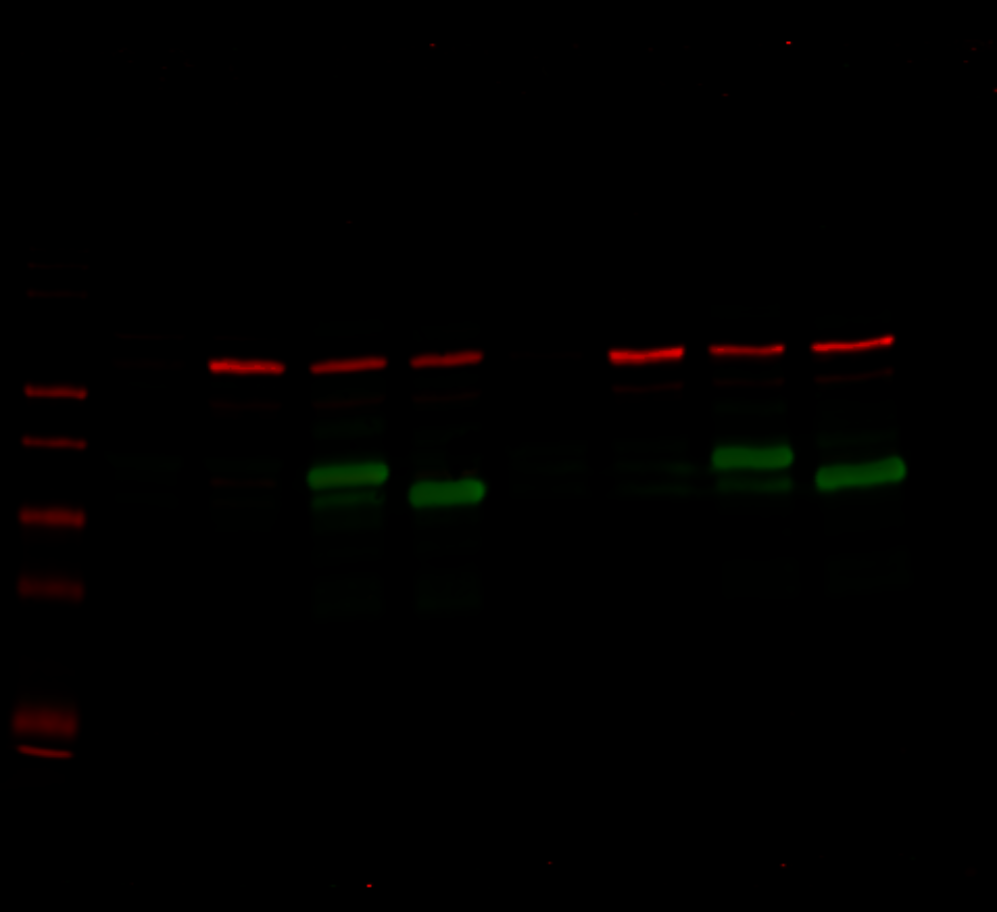

Supplement: Figure 6—source data 6. [file elife-106469-fig6-data6.zip › Figure 6_raw blot/Figure 6A_raw blot/Anti-FLAG-Anti-V5.png]

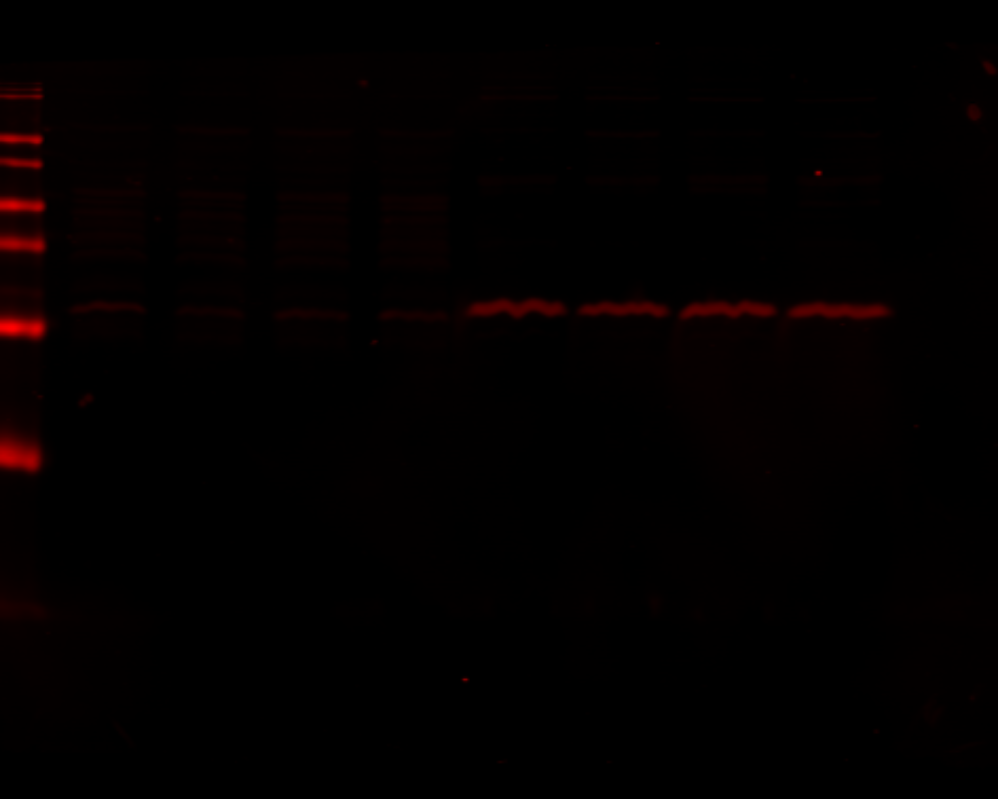

Supplement: Figure 6—source data 6. [file elife-106469-fig6-data6.zip › Figure 6_raw blot/Figure 6A_raw blot/Anti-Histone H3.png]

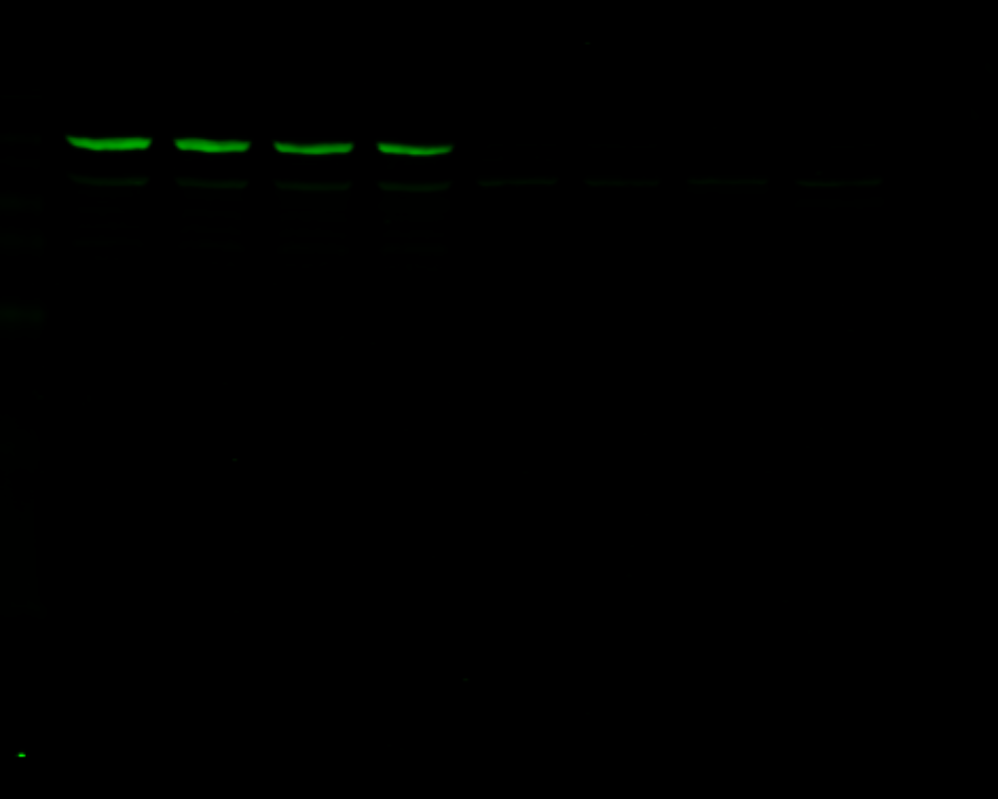

Supplement: Figure 6—source data 6. [file elife-106469-fig6-data6.zip › Figure 6_raw blot/Figure 6A_raw blot/Anti-tubulin.png]

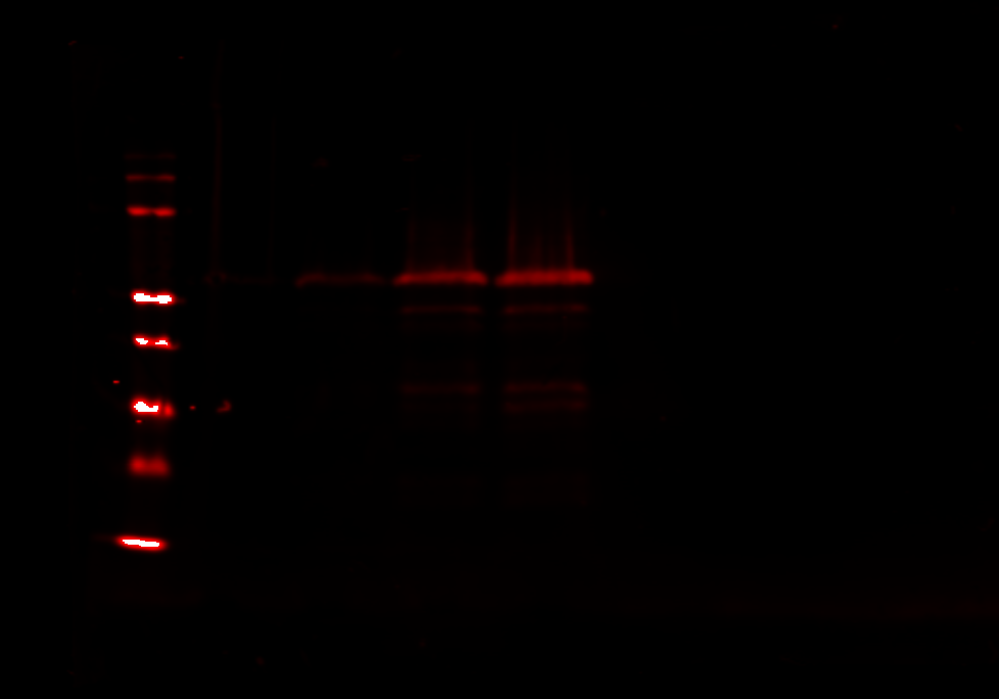

Supplement: Figure 6—source data 6. [file elife-106469-fig6-data6.zip › Figure 6_raw blot/Figure 6B_raw blot/Anti-FLAG.png]

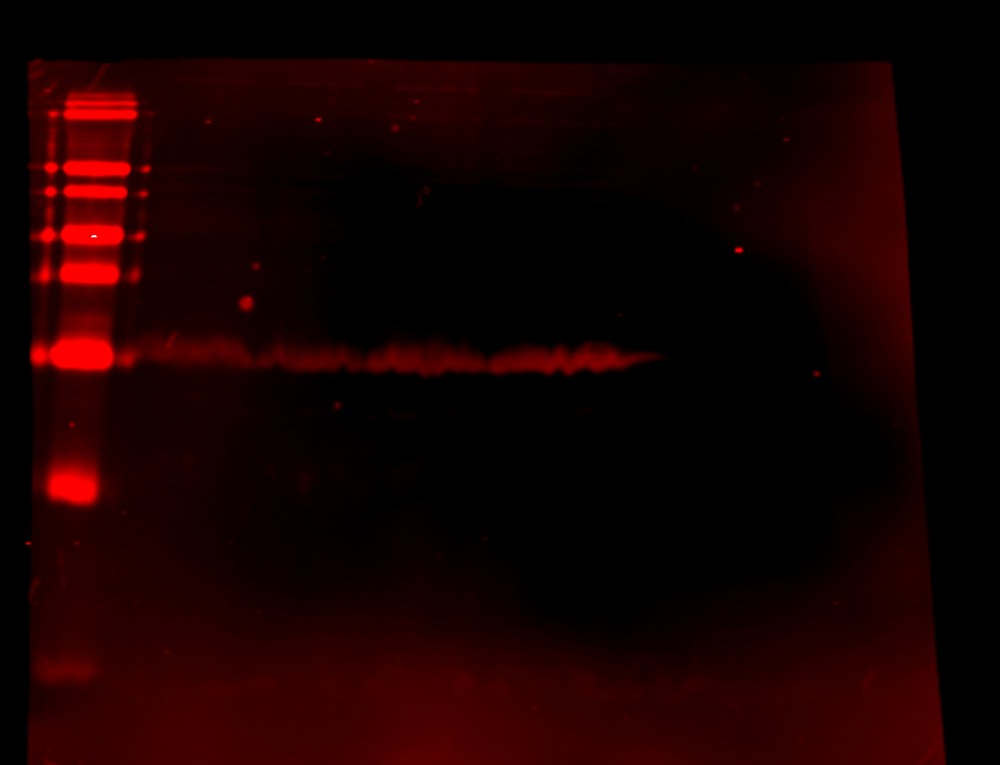

Supplement: Figure 6—source data 6. [file elife-106469-fig6-data6.zip › Figure 6_raw blot/Figure 6B_raw blot/Anti-Histone H3.png]

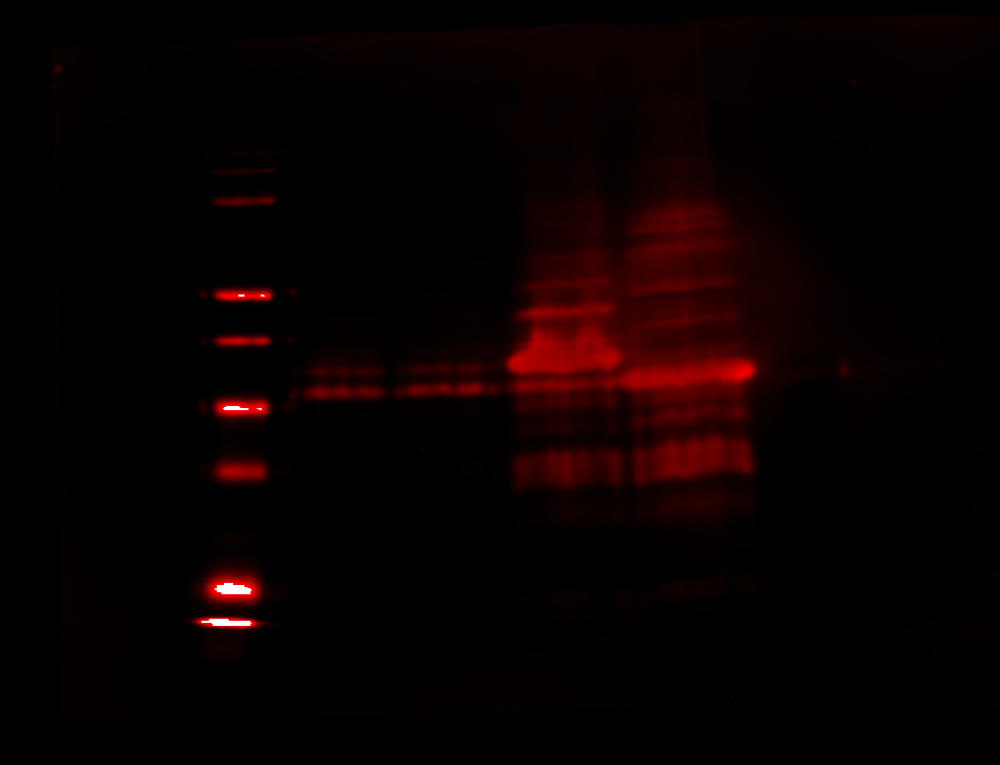

Supplement: Figure 6—source data 6. [file elife-106469-fig6-data6.zip › Figure 6_raw blot/Figure 6B_raw blot/Anti-V5.png]

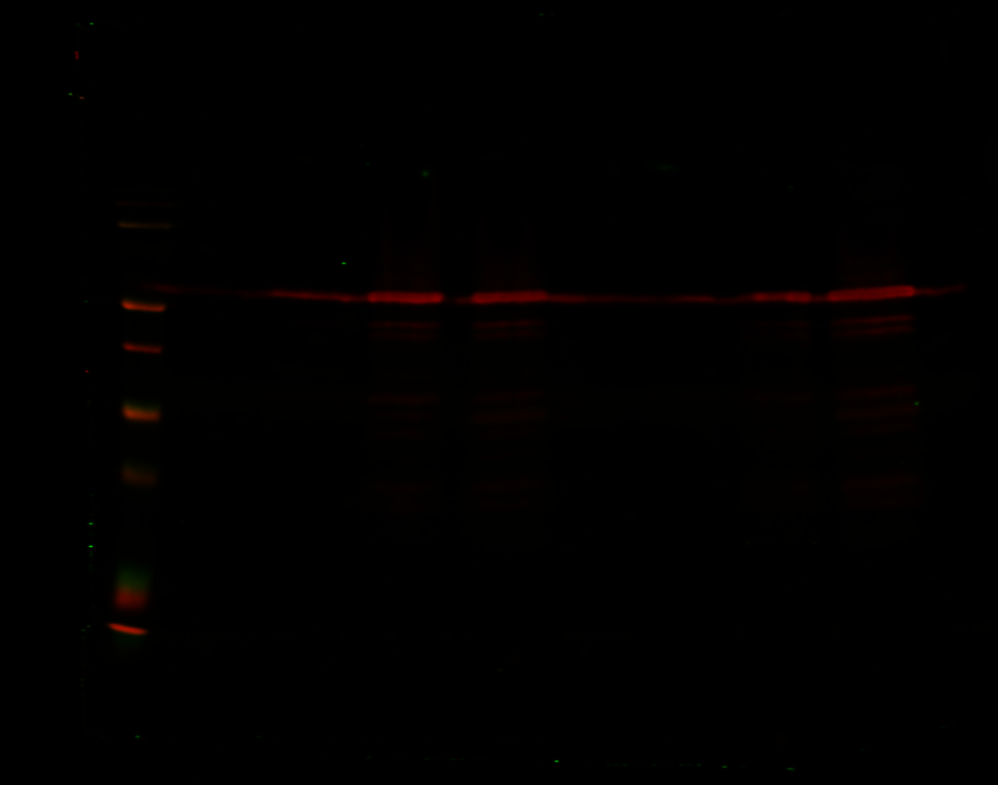

Supplement: Figure 6—figure supplement 1—source data 2. [file elife-106469-fig6-figsupp1-data2.zip › Figure 6—figure supplement 1/Anti-FLAG.png]

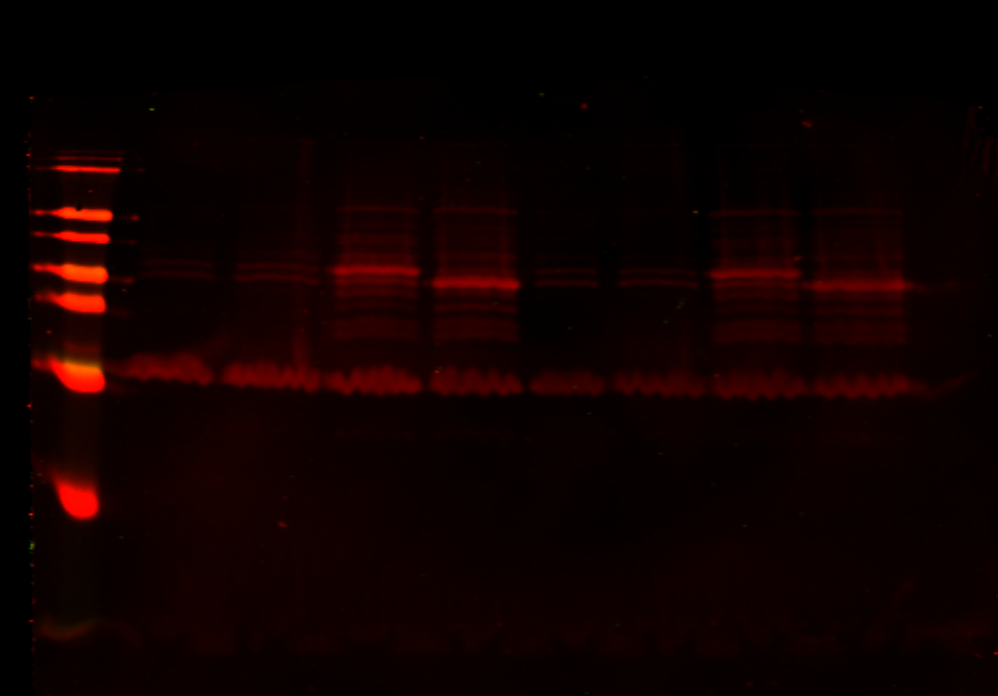

Supplement: Figure 6—figure supplement 1—source data 2. [file elife-106469-fig6-figsupp1-data2.zip › Figure 6—figure supplement 1/Anti-Histone H3.png]

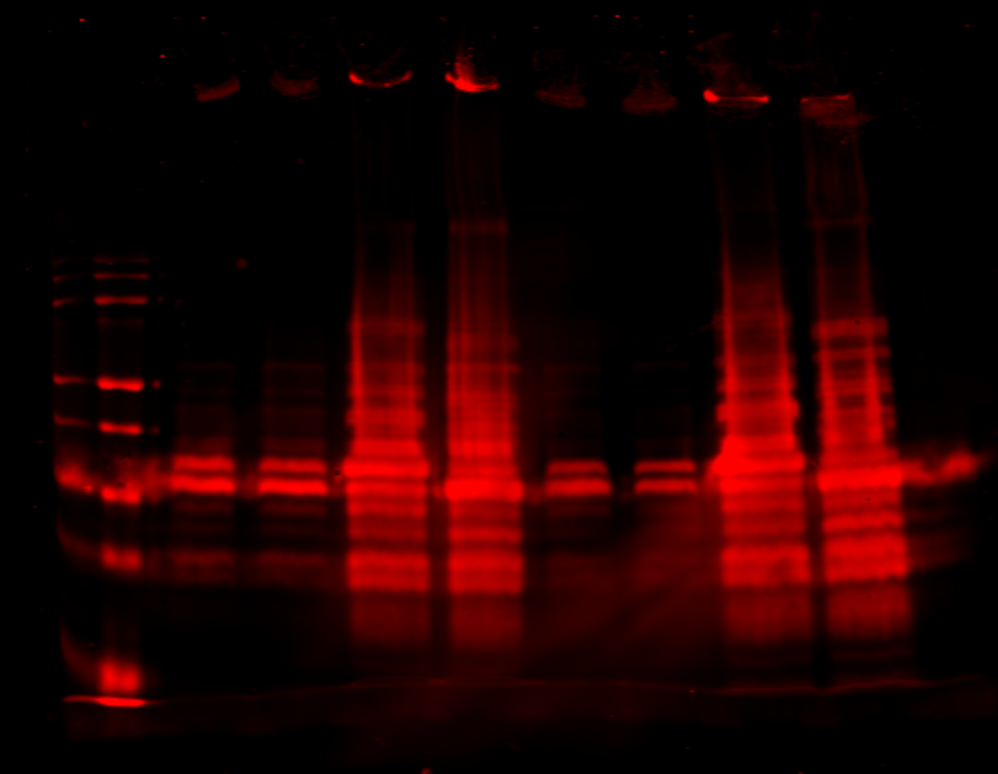

Supplement: Figure 6—figure supplement 1—source data 2. [file elife-106469-fig6-figsupp1-data2.zip › Figure 6—figure supplement 1/Anti-V5.png]

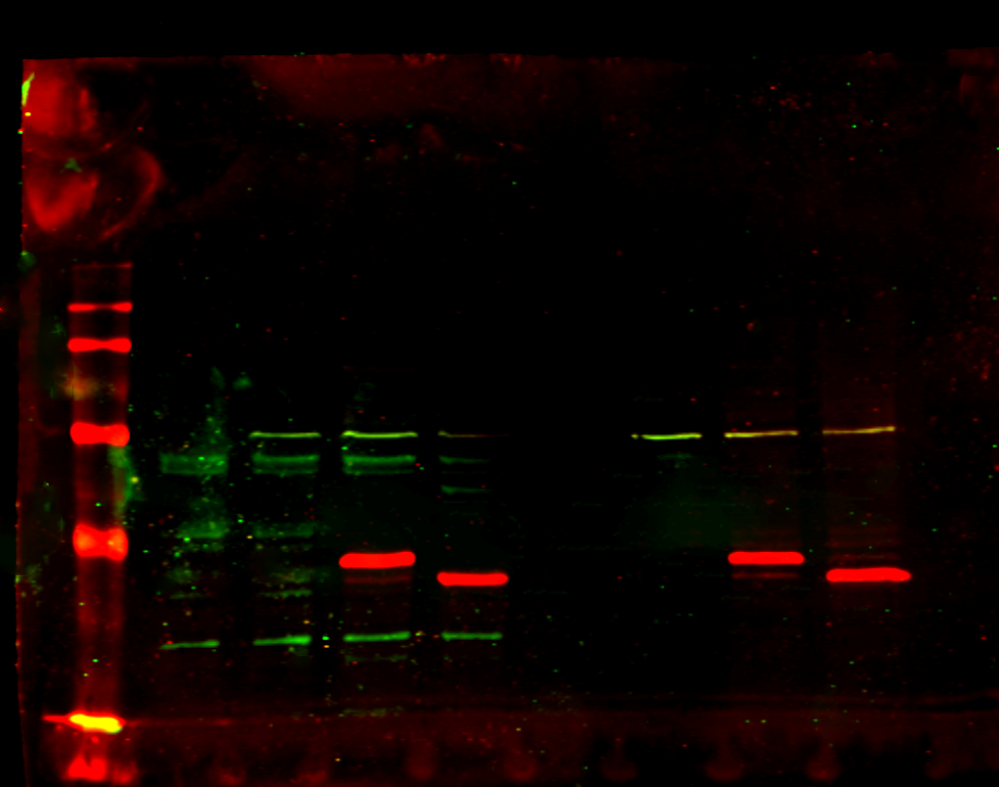

Supplement: Figure 6—figure supplement 2—source data 5. [file elife-106469-fig6-figsupp2-data5.zip › Figure 6—figure supplement 2/Figure 6-figure supplement 2A/Anti-FLAG-Anti-p53 FL-393.png]

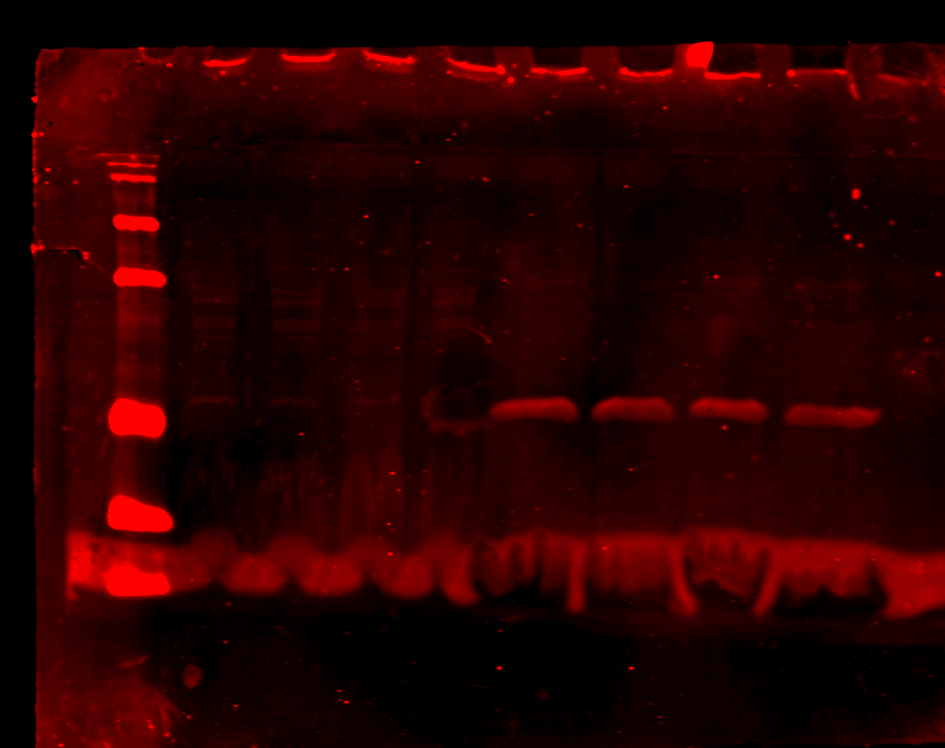

Supplement: Figure 6—figure supplement 2—source data 5. [file elife-106469-fig6-figsupp2-data5.zip › Figure 6—figure supplement 2/Figure 6-figure supplement 2A/Anti-Histone H3.png]

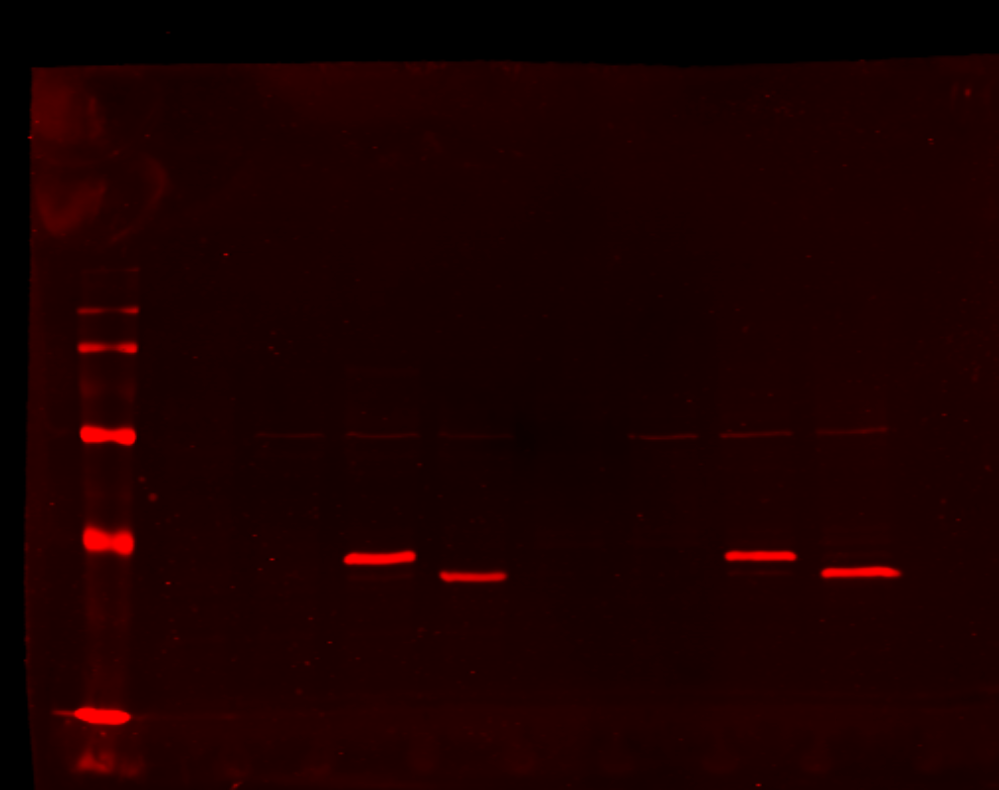

Supplement: Figure 6—figure supplement 2—source data 5. [file elife-106469-fig6-figsupp2-data5.zip › Figure 6—figure supplement 2/Figure 6-figure supplement 2A/Anti-p53 FL-393.png]

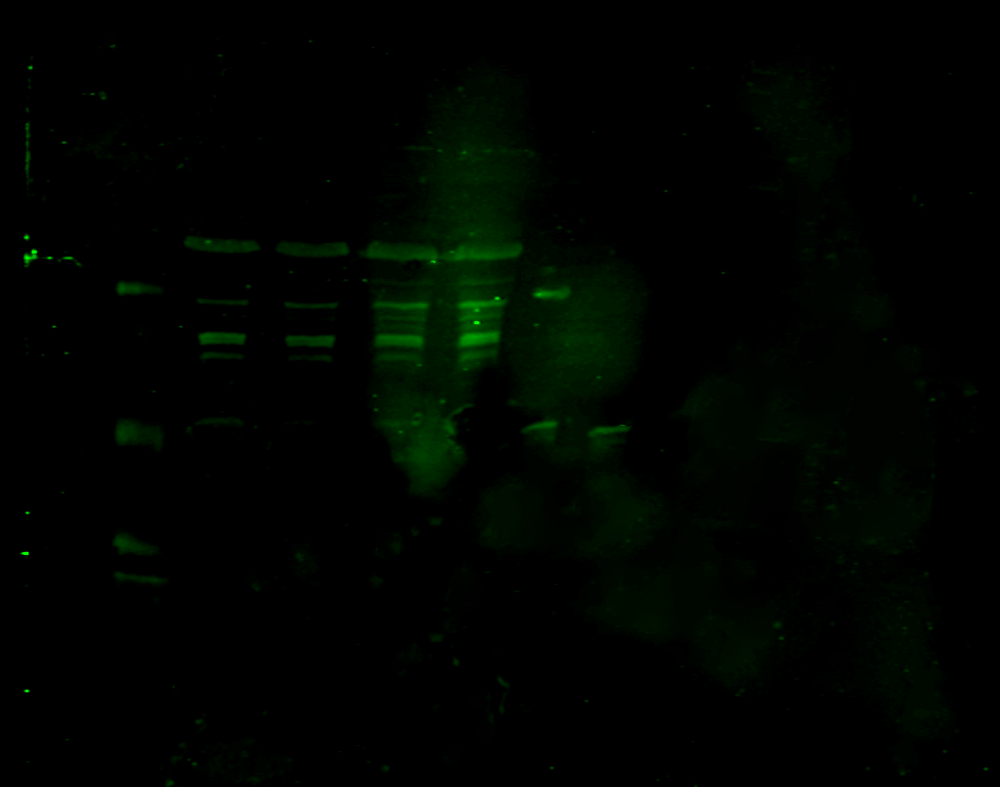

Supplement: Figure 6—figure supplement 2—source data 5. [file elife-106469-fig6-figsupp2-data5.zip › Figure 6—figure supplement 2/Figure 6-figure supplement 2A/Anti-tubulin.png]

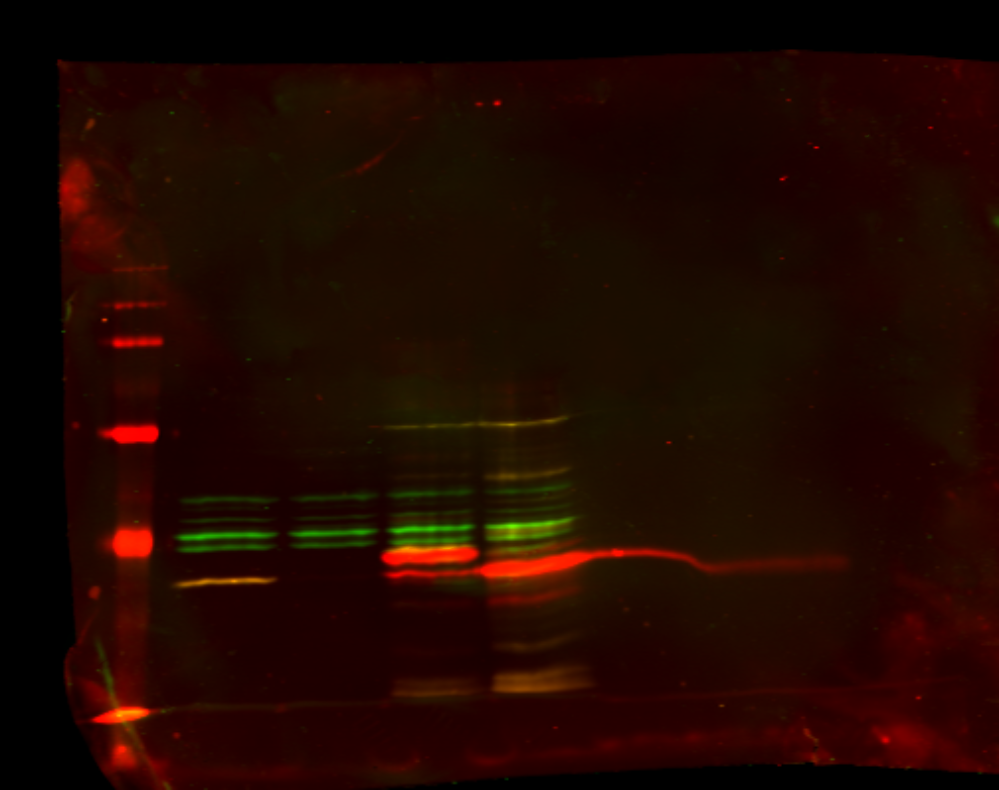

Supplement: Figure 6—figure supplement 2—source data 5. [file elife-106469-fig6-figsupp2-data5.zip › Figure 6—figure supplement 2/Figure 6-figure supplement 2B/Anti-FLAG-Anti-p53 FL-393.png]

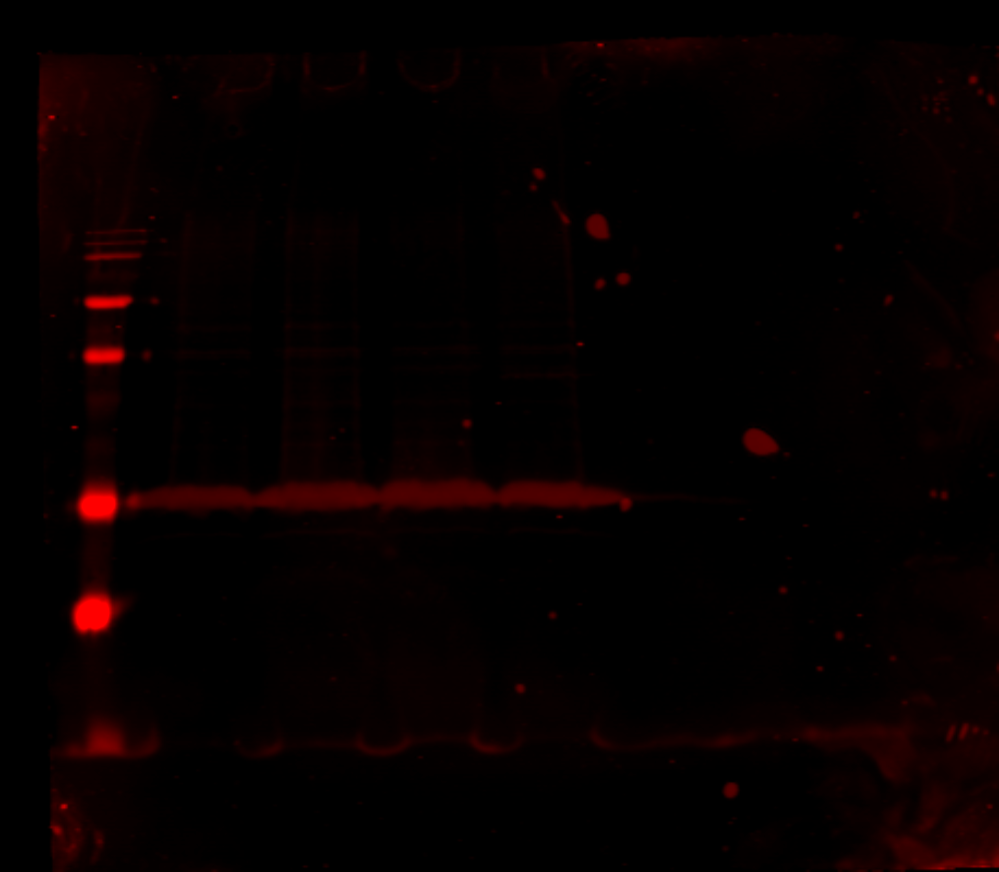

Supplement: Figure 6—figure supplement 2—source data 5. [file elife-106469-fig6-figsupp2-data5.zip › Figure 6—figure supplement 2/Figure 6-figure supplement 2B/Anti-Histone H3.png]

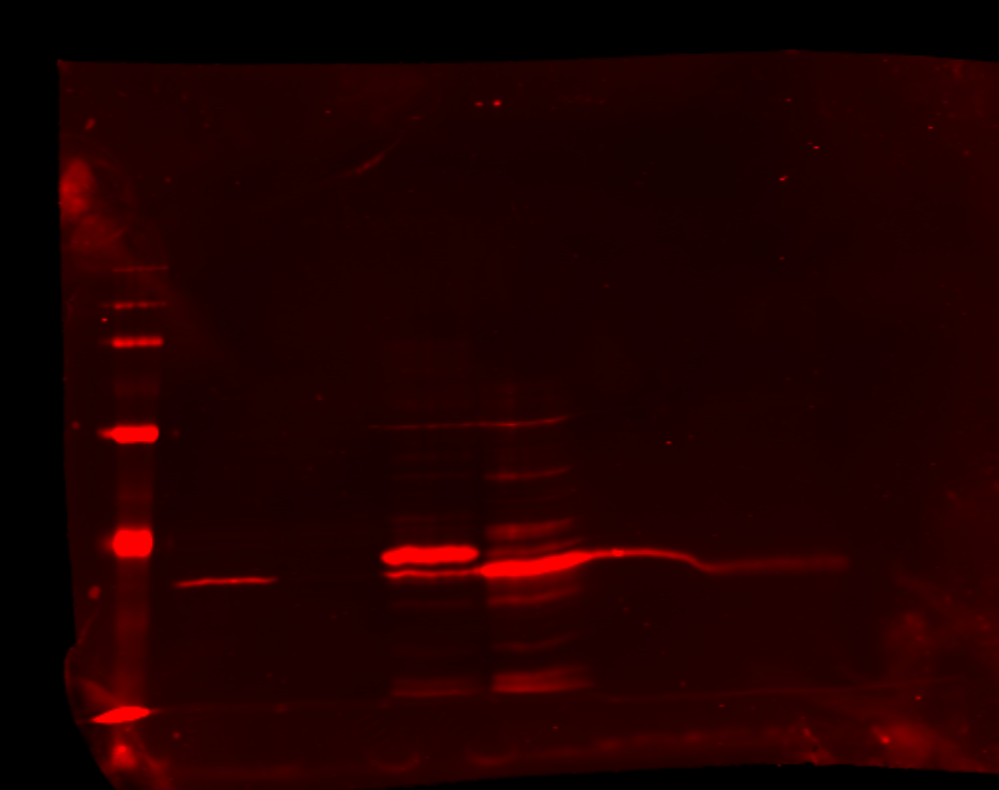

Supplement: Figure 6—figure supplement 2—source data 5. [file elife-106469-fig6-figsupp2-data5.zip › Figure 6—figure supplement 2/Figure 6-figure supplement 2B/Anti-p53 FL-393.png]

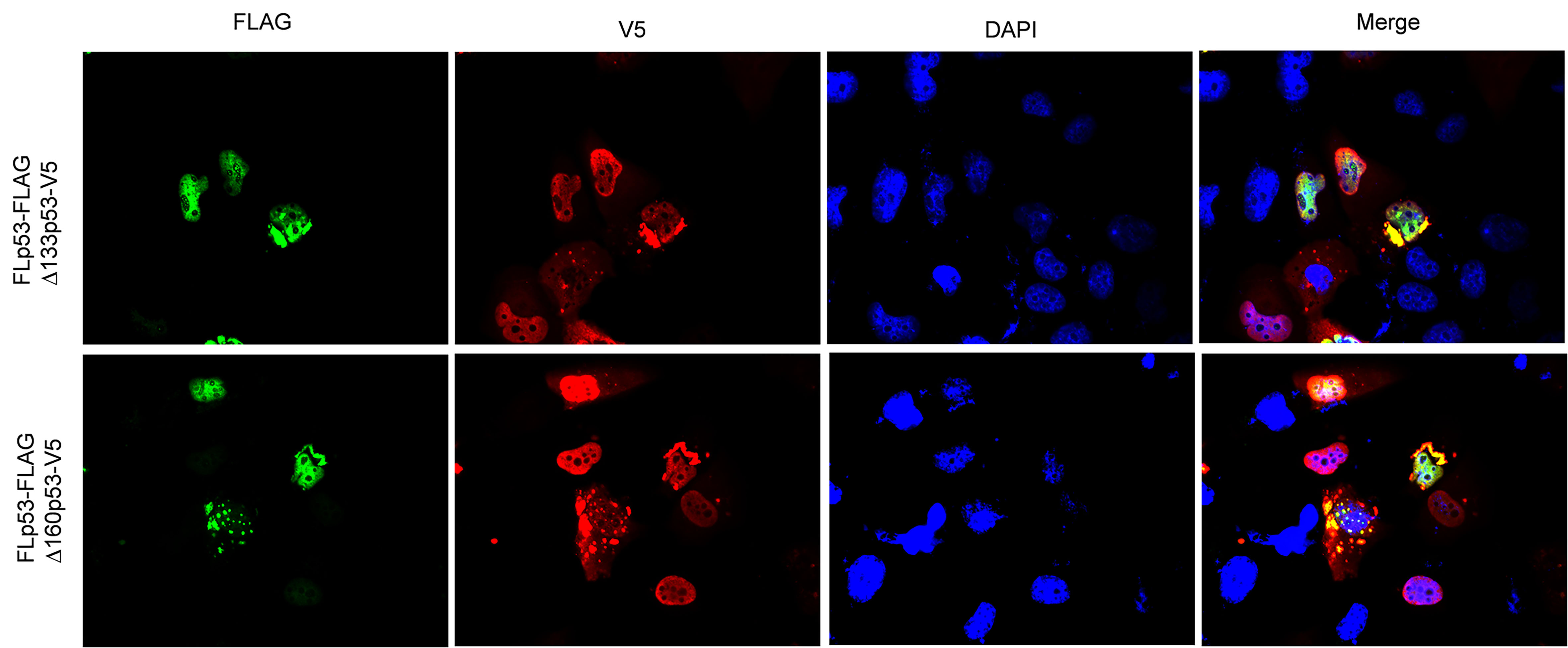

Supplement: Figure 6—figure supplement 3—source data 1. [file elife-106469-fig6-figsupp3-data1.jpg]
