# Supplementary material for: Δ133p53α and Δ160p53α isoforms of the tumor suppressor protein p53 exert dominant-negative effect primarily by co-aggregation
Source: eLife. 2025 Jul 21;14:RP106469. doi: 10.7554/eLife.106469 (PMC12279375; doi:10.7554/eLife.106469)
Supplement: Figure 6—figure supplement 2—source data 2. [file elife-106469-fig6-figsupp2-data2.zip › Figure 6-figure supplement 2-source data 2_labeled blot.pdf]

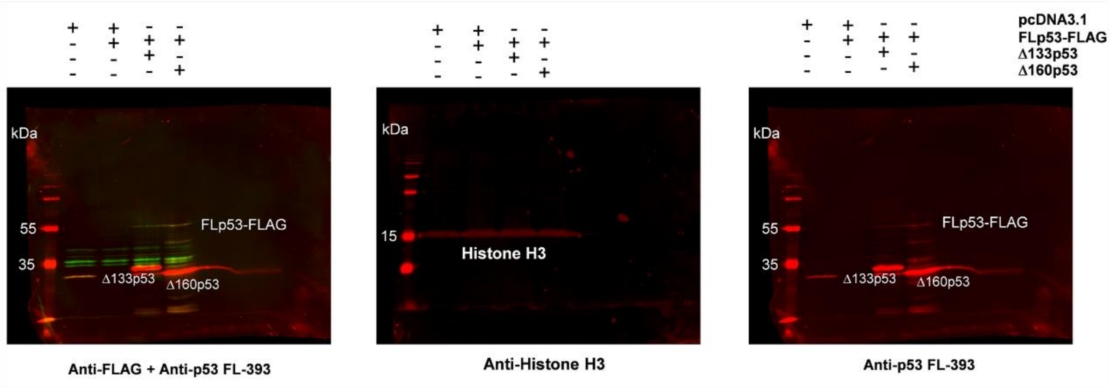

Figure 6-figure supplement 2-source data 2. Original membranes corresponding to Figure 6-figure supplement 2, panel B.
