# Supplementary material for: Δ133p53α and Δ160p53α isoforms of the tumor suppressor protein p53 exert dominant-negative effect primarily by co-aggregation
Source: eLife. 2025 Jul 21;14:RP106469. doi: 10.7554/eLife.106469 (PMC12279375; doi:10.7554/eLife.106469)
Supplement: Supplementary file 1. [file elife-106469-supp1.docx]

## **Supplementary File 1**

Sequences of oligonucleotides used in the work

| **Name** | **Sequence 5' to 3'** | **Description** | **Source** |
| --- | --- | --- | --- |
| **Primers for construction of Δ133p53 and Δ160p53 with FLAG-tag expression plasmids** | | | |
| Δ133p53-FLAG fw | CTAGCTAGCCACCATGTTTTGCCAACTGGCCAAG | Δ133p53- FLAG | This work |
| Δ160p53-FLAG fw | CTAGCTAGCCACCATGGCCATCTACAAGCAGTCA | Δ160p53- FLAG | This work |
| Δ133/160p53-FLAG rev | CCGGAATTCTTATTTATCGTCATCGTC | Δ133/160p53- FLAG | This work |
| **Primers for construction of FLp53 and its two isoforms with V5-tag expression plasmids** | | | |
| FLp53-V5 fw | CCGGAATTCGCCACCATGGAGGAGCC | FLp53-V5 | This work |
| FLp53-V5 rev | CCGCTCGAGGTCTGAGTCAGGCCCTTCTG | FLp53-V5 | This work |
| Δ133p53-V5 fw | CCGGAATTCGCCACCATGTTTTGCCAACTGGCCAAG | Δ133p53-V5 | This work |
| Δ160p53-V5 fw | CCGGAATTCGCCACCATGGCCATCTACAAGCAG | Δ160p53-V5 | This work |
| **Primers for construction of untagged proteins expression plasmids** | | | |
| FLp53 fw | CTAGCTAGCCACCATGGAGGA | FLp53 | This work |
| FLp53 rev | CCGGAATTCTTAGTCTGAGTCAGGCCCTTCT | FLp53 | This work |
| Δ133p53 fw | CTAGCTAGCCACCATGTTTTGCCAACTGGCCAAG | Δ133p53 | This work |
| Δ160p53 fw | CTAGCTAGCCACCATGGCCATCTACAAGCAG | Δ160p53 | This work |
| **Primers for construction of Bax-Luc expression plasmids** | | | |
| baxP fw | CCGCTCGAGGCTTCAGCCCGGGAAT | BAX promoter | This work |
| baxP rev | CCCAAGCTTAGCTCTCCCCAGCGCAGA | BAX promoter | This work |
| **Primers for ChIP-qPCR assay** | | | |
| p21 5`RE fw | AGCAGGCTGTGGCTCTGATT | p53 5`RE of  p21 promoter | (Laptenko et al., 2011) |
| p21 5`RE rev | CAAAATAGCCACCAGCCTCTTCT | p53 5`RE of  p21 promoter | (Laptenko et al., 2011) |
| MDM2 fw | TCAAGTTCAGACACGTTCCGAA | p53 RE of  MDM2 promoter | (Laptenko et al., 2011^)^ |
| MDM2 rev | CTGGGAAAATGCATGGTTTAAATA | p53 RE of  MDM2 promoter | (Laptenko et al., 2011) |
| PUMA fw | TCAGTGTGTGTGTCCGACTGTC | p53 RE of  PUMA promoter | (Laptenko et al., 2011) |
| PUMA rev | GGCAGGGCCTAGCCCA | p53 RE of  PUMA promoter | (Laptenko et al., 2011) |
| Bax fw | AGGCTGAGACGGGGTTATCT | p53 RE of  BAX promoter | This work |
| Bax rev | CAAGTGCAAAAGCTCAGAGG | p53 RE of  BAX promoter | This work |

Reference

Laptenko, O., Beckerman, R., Freulich, E., and Prives, C. (2011). **p53 binding to nucleosomes within the p21 promoter in vivo leads to nucleosome loss and transcriptional activation**. *Proceedings of the National Academy of Sciences of the United States of America* **108:** 10385-10390.
